# Gender development and hepatitis B and C infections among pregnant women in Africa: a systematic review and meta-analysis

**DOI:** 10.1186/s40249-019-0526-8

**Published:** 2019-03-04

**Authors:** Jean Joel Bigna, Angeladine M. Kenne, Aghiles Hamroun, Marie S. Ndangang, Audrey Joyce Foka, Dahlia Noelle Tounouga, Rémi Lenain, Marie A. Amougou, Jobert Richie Nansseu

**Affiliations:** 1 Department of Epidemiology and Public Health, Centre Pasteur of Cameroon, P.O. Box 1274, Yaoundé, Cameroon; 2 Faculty of Medicine, University of Paris Sud, Le Kremlin-Bicetre, France; 3 Department of Nephrology, Huriez Hospital, Lille University Hospital, Lille, France; 4 Department of Medical Information and Informatics, Rouen University Hospital, Rouen, France; 5 Faculty of Medicine and Biomedical Sciences, University of Yaoundé I, Yaoundé, Cameroon; 6 MethodS in Patient-centered outcomes & HEalth ResEarch (EA 4275 SPHERE), Nantes University, Nantes, France; 7 Department of Virology, Centre Pasteur of Cameroon, Yaoundé, Cameroon; 8 Faculty of Sciences, University of Yaoundé I, Yaoundé, Cameroon; 9 Department for the Control of Disease, Epidemics and Pandemics, Ministry of Public Health, Yaoundé, Cameroon; 10 Department of Public Health, Faculty of Medicine and Biomedical Sciences, University of Yaoundé I, Yaoundé, Cameroon

**Keywords:** Hepatitis B, Hepatitis C, Development, Africa, Gender, Pregnant, Women health

## Abstract

**Background:**

Although Africa is a region of hyper endemicity to viral hepatitis B (HBV) and C (HCV) infections, there is limited data on their related burden among pregnant women. The present systematic review and meta-analysis aimed to determine the magnitude of these infections among pregnant women living in Africa and investigate its association with gender-related human development indicators.

**Main text:**

We searched PubMed, Embase, Web of Science, Africa Journal Online, and Global Index Medicus, with no language restriction, to identify observational studies on HBV and HCV infections in pregnant women residing in Africa published from January 1, 2000 until December 31, 2017. Eligible studies reported the prevalence of HBV and/or HCV infection(s) (HBs antigen and HCV antibodies) and/or infectivity (HBe antigen or detectable HCV viral load). Each study was independently reviewed for methodological quality. We used a random-effects model meta-analysis to pool studies. In total, 145 studies (258 251 participants, 30 countries) were included, of which 120 (82.8%) had a low, 24 (16.5%) a moderate, and one (0.7%) had a high risk of bias. The prevalence of HBV and HCV infections was 6.8% (95% confidence interval [*CI*]: 6.1–7.6, 113 studies) and 3.4% (95% *CI*: 2.6–4.2, 58 studies), respectively. The prevalence of HBe antigen and HCV detectable viral load was 18.9% (95% *CI*: 14.4–23.9) and 62.3% (95% *CI*: 51.6–72.5) in HBV positive and HCV positive pregnant women, respectively. The multivariable meta-regression analysis showed that the prevalence of HBV infection increased with decreasing gender development index, males’ level of education and females’ expected years of schooling. Furthermore, this prevalence was higher in rural areas and in western and central Africa. The prevalence of HCV infection increased with decreasing proportion of seats held by women in parliament.

**Conclusions:**

To address the burden of HBV and HCV infections, beyond well-known risk factors at the individual-level, macro-level factors including gender-related human development indicators and dwelling in rural areas should be considered. In Africa, HBV or HCV infected mothers seems to have high potential of transmission to their children.

**Electronic supplementary material:**

The online version of this article (10.1186/s40249-019-0526-8) contains supplementary material, which is available to authorized users.

## Multilingual abstracts

Please see Additional file 1 for translations of the abstract into five official working languages of the United Nations.

## Background

The World Health Organization (WHO) has launched a global programme against hepatitis in May 2016 which aims to reduce by 90% the number of new cases of hepatitis, reduce by 65% the number of hepatitis related deaths, and treat 80% of eligible people infected with viral hepatitis by 2030 [1]. Actually, hepatitis B virus (HBV) and hepatitis C virus (HCV) are among the most common viral infections worldwide. Latest estimates from the WHO reveal that around 325 million people worldwide are living with chronic HBV or HCV infection, most of whom come from East Asia or sub-Saharan Africa [2]. Both infections share similar routes of transmission including contaminated blood product exposure, sexual activity or perinatal transmission.

As a matter of fact, pregnancy in women with chronic HBV or HCV infection is associated with mother-to-child transmission (MTCT); additionally, it has been shown that these pregnancies may be associated with an increased risk of maternal and foetal complications [3, 4]. Therefore, investigating hepatitis B surface antigen (HBsAg) and antibodies against hepatitis C (HCVAb) seroprevalence during pregnancy, and opposing appropriate management are fundamental to prevent MTCT. Moreover, these seroprevalence estimates among pregnant women may constitute a reliable indicator of the prevalence in the general population as well as a determinant of vaccination interventions, among other strategies [4–6].

Although Africa is a region of hyper endemicity to HBV and HCV infections, there is limited data on the burden of these viral infections among pregnant women and consequential MTCT, at the continent level. Such data are mandatory for healthcare planners and caregivers to design and implement evidence-based interventions. Accordingly, it is also important to understand how gender differences can influence the epidemiology of these viral infections in the continent, since there may be a relationship between gender differences and health [7]. Therefore, we present here, the systematic review including a meta-analysis aiming to comprehensively summarize existing knowledge on the prevalence and magnitude of HBV and HCV infections among pregnant women living in Africa. Singularly and using a global approach, we sought to identify gender-related human development indicators likely associated with the increased prevalence of HBV and HCV infections all-round the continent.

## Main text

### Methods

#### Search strategy and selection criteria

We searched PubMed, EMBASE, Web of Science, Africa Journal Online, and Global Index Medicus to identify relevant studies published on HBV and/or HCV among pregnant women living in Africa, published from January 1, 2000 to December 31, 2017, without any language restriction. We limited the search to the last 18 years to have contemporaneous data. The main search strategy for Embase is presented in the Additional file 2: Table S1. This search strategy was adapted to fit with other databases. To supplement these database searches, references of all relevant studies were also screened to identify additional potential data sources. The last search in all electronic databases was conducted on January 1st, 2018.

We considered cross-sectional studies of pregnant women living throughout the African continent, which reported the prevalence of HBV infection (HBsAg) and/or infectivity (HBe antigen [HBeAg]), and/or HCV infection markers (HCVAb and/or HCV detectable viral load), or having enough data to compute these estimates. We excluded case series (< 20 participants), letters, reviews, commentaries, editorials, and studies without primary data even after two unsuccessful requests addressed to the corresponding author. For studies published in more than one report (duplicates), we considered the most comprehensive study that reported the largest sample size.

Two review authors (AMK and JJB) independently screened the titles and abstracts of articles retrieved from the literature search, and all full texts of potentially eligible articles were obtained and further assessed for final inclusion. Disagreements were resolved through discussions between review authors (AMK and JJB) until a consensus was reached, or arbitration by a third review author (JRN).

#### Data extraction and management

Using a pretested data extraction form, four pairs of review authors (AH, AMK, DNT, JJB, AJF, MSN, MAA, and RL) independently extracted relevant information, including first author, year of publication and period of participants’ recruitment, country of recruitment, site, area, setting, timing of data collection, study design, sampling method, sample size, mean or median age, age range, level of education, marital status, history of blood transfusion, exposure to HBV and/or HCV infection(s), proportion of participants with vaccination against HBV, proportion with HIV infection, number of samples tested for HCV and/or HBV, and number of participants with HBsAg, HBeAg, HCVAb, and HCV detectable viral load. Infection was defined as the presence of HBsAg or HCVAb for HBV and HCV, respectively. Infectivity was defined by the presence of both HBsAg and HBeAg for HBV and both HCVAb and detectable viral load for HCV. On the other hand, we reported the prevalence of HBeAg and HCV detectable viral load respectively among HBV and HCV positive pregnant women, to determine the proportion of women who can transmit the virus to their foetus [8]. The United Nations Statistics Division (UNSD) of the African region and human development indicators including human development index, gender development index [9, 10], life expectancy at birth, gross national income per capita, mean years of schooling, proportion of population with at least some secondary education, share of seats in parliament (% held by women), and sex ratio female/male of unemployment rate, were assigned to each study according to the country of recruitment. In the case of multinational studies, the median was reported. Disagreements were reconciled through discussion and consensus between review authors. When relevant data were not available, we directly contacted the corresponding author of the study to request the information, at least on two different attempts.

We assessed the methodological quality of included studies using an adapted version of the risk of bias tool for prevalence studies, which was developed by Hoy and colleagues [11]. Four pairs of review authors (AH, AMK, DNT, JJB, AJF, MSN, MAA, and RL) independently assessed study methodological quality, with disagreements resolved by consensus or arbitration by a third author (JRN). The total score ranged from 0 to 10 with the overall score categorized as follows: 8–10: “low risk”, 5–7: “moderate risk”, and 0–4: “high risk” of bias.

#### Data synthesis and analysis

We analysed our data using the packages ‘meta’ of R (version 3.5.1). We calculated unadjusted prevalence on the basis of crude numerators and denominators provided by individual studies. To keep the effect of studies with extremely small or extremely large prevalence estimates on the overall estimate to a minimum, we stabilized the variance of the study-specific prevalence using the Freeman–Tukey double arc-sine transformation before pooling data with the random-effects meta-analysis model [12]. Following the crude overall prevalence, we conducted two sensitivity analyses to assess the robustness of our findings including only studies with low risk of bias and excluding studies which reported only HIV-infected pregnant women. Heterogeneity across included studies was assessed using the *χ*^2^ test, and quantified using the H and *I*^2^ statistics [13]. When substantial heterogeneity was detected (*I*^2^ > 50%) [14], we performed subgroup and meta-regression analyses to investigate the possible sources of heterogeneity. We conducted a multivariable meta-regression analysis by including study-level factors that were significant (at *P* <  0.25) in the univariable model. For categorical variables, the global *P* value was considered for inclusion in multivariable models. We applied a manual backward selection procedure to identify factors independently associated with the variation of overall prevalence and successively removed from the model the less significantly associated variables. We reported the explained heterogeneity (*R*^2^) by variables included in models. A *P* value < 0.05 was considered statistically significant. For each of the study-level characteristics, we compared different proportions of potential subgroups across studies (UNSD regions and areas). We used the symmetry of funnel plots and carried-out the Egger test to assess presence of publication and selective reporting bias [15]. A *P* value < 0.10 on the Egger test was indicative of publication bias. It was decided a priori that if publication bias were present it would not be adjusted for, since we believe that the prevalence estimates of interest would likely be published even if substantially different from previously reported estimates. Inter-rater agreements between investigators for study inclusion and methodological quality assessment were assessed using Cohen’s κ [16].

This systematic review is registered in the PROSPERO International Prospective Register of systematic reviews, registration number CRD42018085609.

## Results

### Review process

**Fig. 1:**
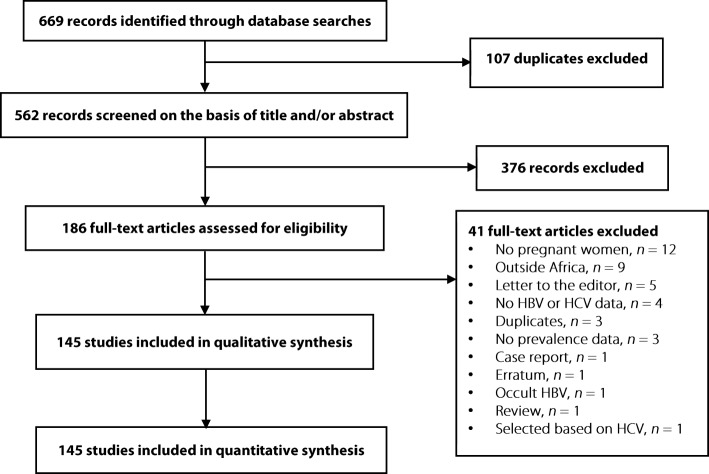
Selection of studies. HBV: Hepatitis B virus; HCV: Hepatitis C virus

We initially identified 669 records; after elimination of duplicates, 562 records remained. We screened their titles and abstracts, and excluded 376 irrelevant records. Agreement between investigators on abstract selection was κ = 0.73. We scrutinised full texts of the remaining 186 papers for eligibility, of which 41 papers were excluded. Finally, as presented in Fig. 1 and Additional file 2: Table S2, a total of 145 full texts - including 87 for HBV, 33 for HCV, and 25 for both infections - were retained. The inter-rater agreement between investigators was κ = 0.92 for study final inclusion and varied from 0.52 to 0.97 for data extraction (all *P* values < 0.05).

### Characteristics of included studies

Table 1 summarizes characteristics of included studies. Overall, 145 studies involving 208 251 participants from 30 countries (Additional file 2: Table S3) were included in this study. Most studies were from the western region of Africa (54.5%), were prospective (88.3%), single-site (64.8%), conducted in antenatal care settings (74.6%), and used consecutive sampling (78.6%). Individual characteristics of included studies are presented in Additional file 2: Table S4. One hundred and twenty studies (82.8%) had a low risk of bias, 24 (16.5%) had a moderate risk and one study (0.7%) had a high risk of bias.

**Table 1:** Characteristics of included studies

Characteristics	Description
Participants’ characteristics	
Mean or median age in year, range (*n* = 95)	23–34.1
Age range in years (*n* = 142)	10–55.0
% of vaccinated against HBV, range (*n* = 21)	0.0–8.9
% with secondary level of education or higher, range (*n* = 47)	0.5–100
Marital status, %	
- Single (*n* = 15)	0.0–37.6
- Married (*n* = 51)	2.7–100
- Divorced (*n* = 31)	0.0–12.9
- Widow (*n* = 27)	0.0–16.7
% in polygamous marriage (*n* = 16)	2.8–56.0
% of ever received blood transfusion, range (*n* = 58)	0.0–45.0
% living in rural, range (*n* = 21)	0.0–100.0
% with history of multiple sexual partners, range (*n* = 84)	0.0–54.0
% with history of any surgery procedure, range (*n* = 39)	0.5–65.4
% with any dental procedure, range (*n* = 16)	3.1–80.8
% with the first pregnancy, range (*n* = 51)	2.6–73.0
% with scarification, range (*n* = 21)	9.0–89.2
% with tattoos, range (*n* = 28)	0.0–64.5
% with excision, range (*n* = 14)	0.0–96.7
% with history of abortion, range (*n* = 23)	0.0–63.0
% with history of traditional birth attendant, range (*n* = 4)	8.0–50.0
Studies’ characteristics	
Period of inclusion, range	1995–2017
Region, *n* (%)	
- Western	79 (54.5)
- Eastern	25 (17.3)
- Northern	20 (13.8)
- Middle	14 (9.6)
- Southern	7 (4.8)
Number of sites, *n* (%)	
- Single	94 (64.8)
- Multisite	51 (35.2)
Timing, n (%)	
- Prospective	128 (88.3)
- Retrospective	17 (11.7)
Setting, (%)	
- Antenatal care	108 (74.6)
- Other hospital-based	26 (17.9)
- Antenatal care and other hospital-based	11 (7.6)
- Antenatal care and community-based	1 (0.7)
- Community-based	1 (0.7)
Sampling method, *n* (%)	
- Consecutive	114 (78.6)
- Random	20 (13.8)
- Systematic	11 (7.6)
Human development indicators, median (1st–3rd quartiles)	
Gender development index (*n* = 143)	0.853 (0.847–0.884)
Human development index (*n* = 143)	
- Females	0.482 (0.474–0.531)
- Males	0.569 (0.535–0.577)
Life expectancy at birth, years	
- Females	59.5 (53.4–65.5)
- Males	55.5 (52.7–62.7)
Gross national income per capita, USD (*n* = 144)	
- Females	4132 (1624–4132)
- Males	6706 (2576–6706)
Expected years of schooling, years	
- Females	9.2 (9.2–10.9)
- Males	10.8 (10.5–11.7)
Mean years of schooling (*n* = 14)	
- Females	4.9 (4.0–5.7)
- Males	7.1 (6.2–7.4)
Population with at least some secondary education (% ages 25 and older) (*n* = 144)	
- Females	41.4 (16.4–41.4)
- Males	61.3 (30.5–76.2)
Share of seats in parliament (% held by women)	9.4 (5.8–27.1)
Unemployment rate, female to male ratio	1.2 (1.2–1.5)

*USD* United States Dollar

### Prevalence of HBV and HCV infections and infectivity

**Fig. 2:**
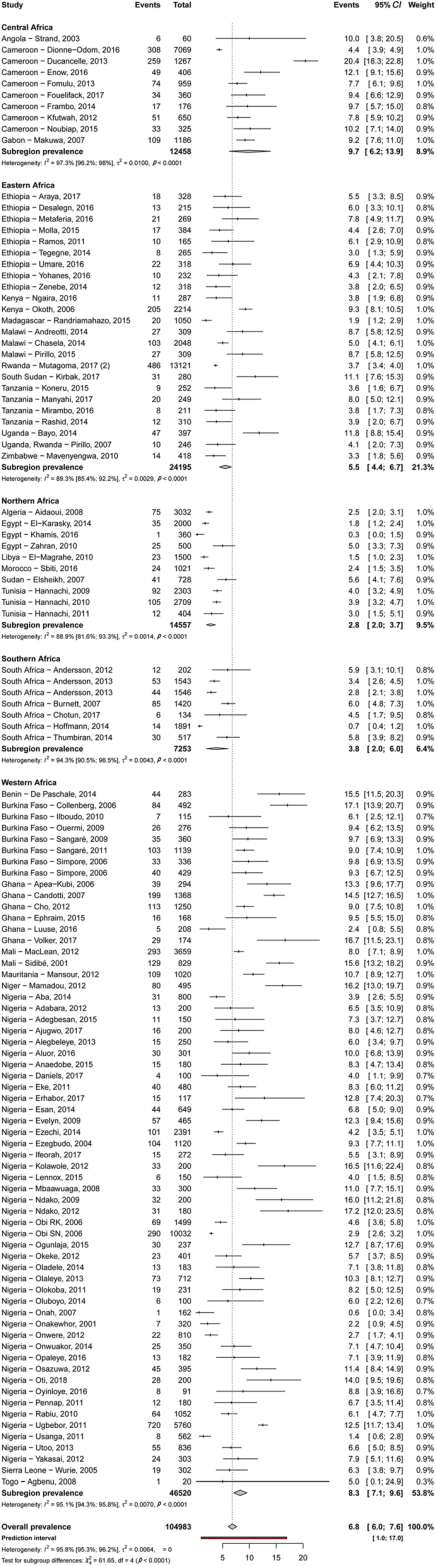
Meta-analysis prevalence of hepatitis B virus infection in pregnant women in Africa. *CI*: Confidence interval; df: Degree of freedom

**Fig. 3:**
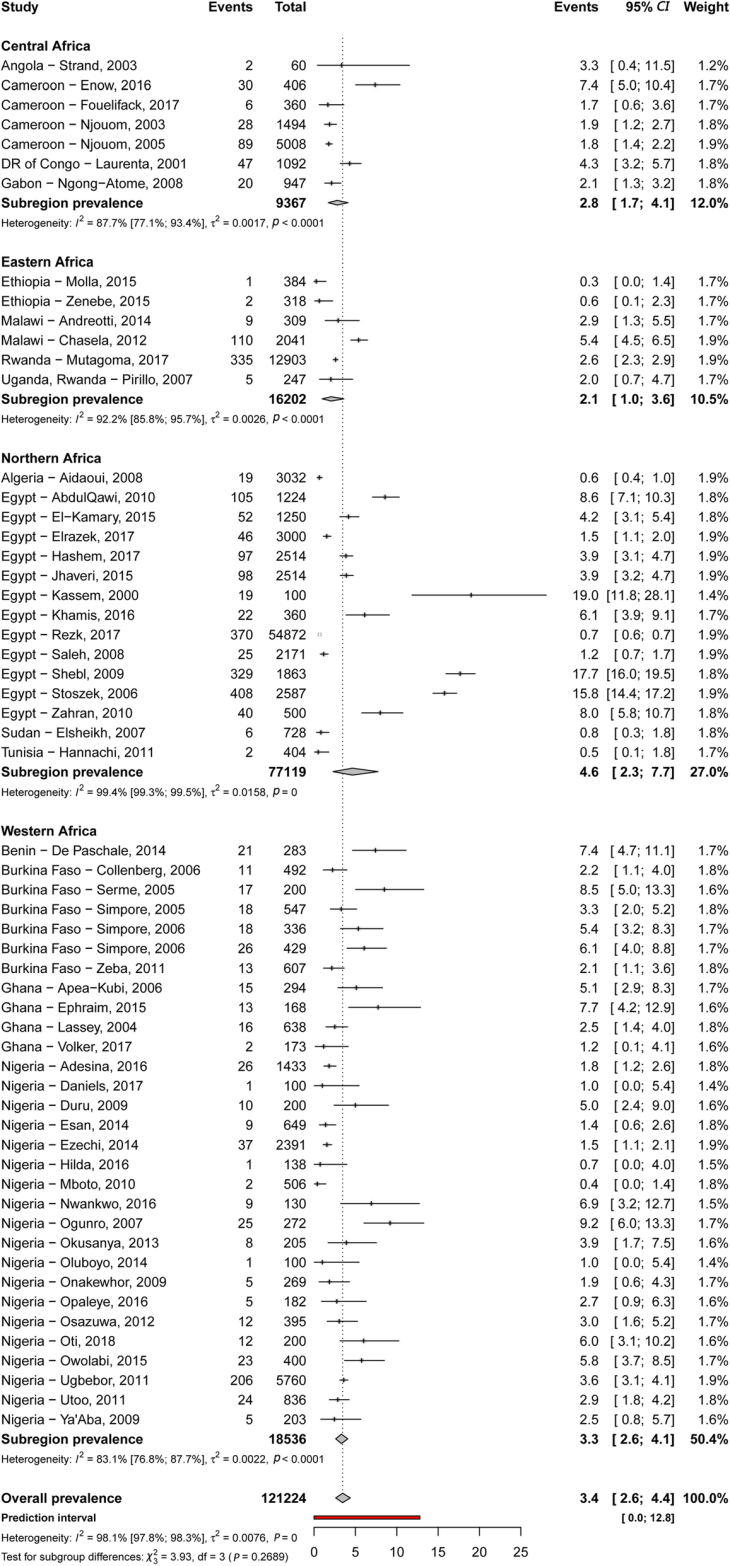
Meta-analysis prevalence of hepatitis C virus infection in pregnant women in Africa. *CI*: confidence interval; df: Degree of freedom

Table 2 summarizes results of the meta-analysis. The HBV and HCV infections’ prevalence varied widely across countries, from 1.5% in Libya to 16.2% in Niger and from 0.4% in Ethiopia to 7.4% in Benin, respectively (Additional file 2: Figures S1 and S2). The overall prevalence of HBV and HCV infections was respectively 6.8% (95% confidence interval [*CI*]: 6.1–7.6) (Fig. 2) and 3.4% (95% *CI*: 2.6–4.4) (Fig. 3), with substantial heterogeneity between studies. Low risk of bias and HIV-excluded sensitivity analyses were close to the main overall analyses (Table 2). Funnel plots for HBV (Additional file 2: Figure S3) and HCV (Additional file 2: Figure S4) prevalence estimates suggested publication bias confirmed by the Egger test (Table 2). Looking at the proportion of potentially infective mothers, the prevalence of HBe antigen and HCV detectable viral load was 18.9% (95% *CI*: 14.4–23.9) and 62.3% (95% *CI*: 51.6–72.5) in already HBV positive and HCV positive pregnant women, respectively.

**Table 2:** Summary statistics of HBV and HCV infections prevalence among pregnant women in Africa

	Prevalence, (95% *CI*)	95% Predictive interval	*N* studies	*N* participants	H (95% *CI*)	*I*^2^ (95% *CI*)	*P* heterogeneity	*P* egger	*P* difference
Hepatitis B									
- Overall	6.8 (6.1–7.6)	0.9–17.5	113	104 983	4.8 (4.6–5.1)	95.7 (95.2–96.1)	< 0.0001	0.0007	–
- Low risk of bias	7.0 (6.1–7.8)	0.9–17.9	94	79 592	5.7 (5.4–6.0)	96.9 (96.5–97.2)	< 0.0001	0.008	–
- HIV excluded	7.0 (6.2–7.9)	0.9–17.8	103	85 432	5.5 (5.3–5.8)	96.7 (96.4–97.1)	< 0.0001	0.007	–
- Antigen HBe in HBV positive	19.0 (14.6–23.8)	1.6–49.1	38	1891	2.4 (2.1–2.7)	82.4 (76.6–86.8)	< 0.0001	0.146	–
By region									
- Central	9.7 (6.2–13.9)	0.4–28.4	10	12 458	5.8 (4.9–6.8)	97.0 (95.9–97.8)	< 0.0001	0.088	< 0.0001
- Western	8.3 (7.1–9.5)	1.4–19.8	62	46 520	4.5 (4.2–4.9)	95.1 (94.3–95.8)	< 0.0001	0.026	
- Eastern	5.5 (4.4–6.7)	1.4–11.9	24	24 195	2.7 (2.2–3.2)	86.0 (80.1–90.2)	< 0.0001	0.063	
- Southern	3.8 (2.0–6.0)	0.0–13.6	7	7253	4.2 (3.2–5.4)	94.2 (90.5–96.5)	< 0.0001	0.334	
- Northern	2.8 (2.0–3.7)	0.5–6.6	10	14 557	3.0 (2.3–3.8)	88.9 (81.6–93.3)	< 0.0001	0.877	
By area									
- Rural	12.2 (9.7–14.8)	4.2–23.1	12	9768	3.1 (2.5–3.8)	89.4 (83.5–93.2)	< 0.0001	0.722	< 0.0001
- Urban	6.0 (4.5–7.7)	0.5–16.4	25	16 833	4.0 (3.6–4.6)	93.9 (92.2–95.3)	< 0.0001	0.0008	
- Both	6.4 (5.6–7.2)	1.3–14.6	76	78 382	4.3 (4.0–4.6)	94.5 (93.6–95.2)	< 0.0001	0.0004	
Hepatitis C									
- Overall	3.4 (2.6–4.4)	0.0–12.8	58	121 224	7.1 (6.7–7.5)	98.0 (97.8–98.2)	< 0.0001	0.0002	–
- Low risk of bias	3.2 (2.4–4.0)	0.0–11.0	49	111 134	6.5 (6.0–6.9)	97.6 (97.3–97.9)	< 0.0001	0.0003	–
- HIV excluded	3.5 (2.6–4.5)	0.0–13.3	54	115 050	7.2 (6.8–7.7)	98.1 (97.9–98.3)	< 0.0001	0.0003	–
- Detectable HCV viral load in HCV positive	62.3 (51.6–72.5)	6.5–100.0	19	1897	5.8 (5.2–6.6)	97.1 (96.3–97.7)	< 0.0001	0.742	–
By regions									
- Northern	4.6 (2.3–7.7)	0.0–22.9	15	77 119	13.0 (12.0–14.1)	99.4 (99.3–99.5)	< 0.0001	0.012	0.269
- Western	3.3 (2.6–4.1)	0.6–7.8	30	18 536	2.4 (2.0–2.8)	82.6 (76.2–87.3)	< 0.0001	0221	
- Central	2.7 (1.7–4.1)	0.2–8.0	7	9367	2.8 (2.1–3.9)	87.7 (77.1–93.4)	< 0.0001	0.190	
- Eastern	2.1 (1.0–3.6)	0.0–8.9	6	16 202	3.3 (2.5–4.4)	90.8 (83.6–94.8)	< 0.0001	0.734	
- Southern	NA	NA	0	NA	NA	NA	NA	NA	
By area									
- Rural	5.0 (1.5–10.2)	0.0–30.8	7	10 813	9.0 (7.7–10.5)	98.8 (98.3–99.1)	< 0.0001	0.801	0.695
- Urban	3.3 (2.0–4.9)	0.0–13.9	22	80 909	8.3 (7.6–9.0)	98.5 (98.3–98.8)	< 0.0001	0.004	
- Both	3.2 (2.4–4.1)	0.2–9.0	29	29 502	3.5 (3.0–3.9)	91.7 (89.2–93.6)	< 0.0001	0.342	

*HBV* Hepatitis B virus, *HCV* Hepatitis C virus, *CI* Confidence interval, *NA* Not applicable

### Subgroups and meta-regression analyses

Table 2 presents subgroup analyses. The prevalence of HBV infection was significantly higher in central (9.7%) and western (8.3%) Africa compared to other regions (eastern 5.5%, southern 3.8%, northern 2.8%) (*P* <  0.0001) (Fig. 2). The prevalence of HBV infection was significantly higher in rural areas (12.2%) compared to urban areas (6.0%) (*P* <  0.0001) (Additional file 2: Figure S5). Regarding HCV infection, there was no difference between regions (*P* = 0.269) (Fig. 2) or areas (*P* = 0.695) (Additional file 2: Figure S6).

The multivariable meta-regression analysis showed that the prevalence of HBV infection significantly increased with decreasing gender development index (*R*^2^ = 6.8%), males’ level of education (*R*^2^ = 0.1%), and females’ expected years of schooling (*R*^2^ = 19.7%), and increased with increasing gender inequality index (*R*^2^ = 30.6%). Furthermore, this prevalence was higher in rural settlements (*R*^2^ = 24.8%), and in western and middle Africa (*R*^2^ = 13.8%). The final model explained 53.8% of the total 88.9% residual heterogeneity (Additional file 2: Table S5). The prevalence of HCV infection increased only with decreasing proportion of seats held by women in parliament. The final model explained 0.95% of the total 96.5% residual heterogeneity (Additional file 2: Table S6).

## Discussion

This systematic review and meta-analysis of 145 studies involving 208 251 pregnant women from 30 countries of Africa found a relatively high prevalence of HBV (6.8%) and HCV (3.4%) infections, with substantial heterogeneity between studies. There was an association between high prevalence of HBV and HCV infections on one hand, and low levels of some human development indicators both from males and females on the other hand. The prevalence of HBV infection was higher in central and western Africa and in rural areas, unlike HCV infection with which there was no significant association. The residual heterogeneity observed in the variation of HBV prevalence was more explained by macro-level gender-related human development indicators (53.8%) compared to HCV prevalence (0.95%).

Recent estimates reported in 2015 demonstrated that the global prevalence of HBV infection in the general population was 3.5%. With the heterogeneity in the distribution between regions, this prevalence was the highest in Western Pacific (6.2%) and African (6.1%) regions [2]. The prevalence found in our study among pregnant women - usually classified as a low risk group for HBV infection - was close to that of the general population in the continent and was in the range driven from a narrative systematic review by Gasim and colleagues (2.4–13%) [17]. This finding supports previous thoughts indicating that viral hepatitis seroprevalence estimates among pregnant women may be a true reflect of the prevalence in the general population [4]. Contrariwise, the prevalence of HCV infection found in this study was higher compared to that in the general population (1, 95% uncertainty interval 0.7–1.6) and was in the range of that reported by a recent narrative review among pregnant women in Africa (1–16.5%) and close to the finding of another review conducted in sub-Saharan Africa (2.5, 95% *CI*: 1.5–4.3) [2, 5, 17]. Given infrequency of intravenous drug use in Africa, exposure of pregnant women to unsafe medical (surgery procedures like caesarean and blood transfusion) and non-medical procedures and behaviours (traditional birth attendance, excision, history of abortion, tattoos, multiple sexual partners) may explain the difference in HCV prevalence between pregnant women and the general public [18]. Indeed, some practices known at elevated risk for HCV infection such as unsafe excision reached 96.7% in some studies included in this review.

The prevalence of HBV infection was higher in central and western Africa, and in rural areas as well. Is exposure to HBV through risky behaviours be higher in central and western Africa than in the rest of the continent or is this related to weak health systems? Western and central Africa are also regions with the weakest HIV infection control indicators [19]. Further studies are needed to understand why these regions seem to have higher HBV prevalence estimates than in the rest of the continent. The highest prevalence in rural areas may be explained by the low level of education in rural compared to urban zones; this low level of education may lead to low awareness of the risks for HBV infection [20, 21]. This may be also explained by higher proportions of excision and traditional birth attendance in rural areas. This finding underlines the need for specific interventions targeting rural areas in the continent. There was an equal distribution of HCV infection throughout regions and areas. Madhava and colleagues who worked on the general population found that the prevalence of HCV infection was higher in central Africa, and in blood donor populations of sub-Saharan Africa [18]. This comparison between the two sets of results reinforces the thought of the heterogeneity regarding the distribution of HCV infection between pregnant women and the general public. Accordingly, further studies are warranted to elucidate this issue.

We found a high prevalence of HBeAg (18.9%) among HBsAg positive women and a high prevalence of HCV detectable viral load (62.3%) among HCV positive pregnant women. Using an empirical Bayesian hierarchical model, Ott and colleagues found that the global HBeAg prevalence varied between 20 and 50% at reproductive ages in women and that this prevalence was between 16 and 43% in Africa [6], in line with our results. The current evidence suggests that the MTCT of HCV is proportional to the HCV viral load, although no transmission of HCV by breastfeeding has been described yet [18]. MTCT of HBV is reported to be responsible for more than one third of chronic HBV infections, globally [22]. Foetuses and neonates with mothers positive to HBV or HCV remain at high risk of infection in Africa. This risk increases in the presence of co-infection with HIV, even more since as Africa has the highest HIV-infection prevalence worldwide [23]. Therefore, implementing strategies to reduce vertical transmission of HBV and HCV infections, including third-trimester antiviral prophylaxis, immunoglobulins at birth, and HBV birth-dose vaccination of new-borns are needed [23, 24]. Such strategies are urgently needed since only about twenty of fifty-five countries in Africa have already implemented or planned an implementation of HBV birth-dose vaccination [25].

In this review, the prevalence of HBV infection increased with decreasing gender development index, decreasing males’ level of education, decreasing females’ expected years of schooling, and increasing gender index inequality. The gender development index is considered as a distribution-sensitive measure that accounts for the human development impact of existing gender gaps in the three components (life expectancy, adult literacy and education, and gross domestic income per capita) of the human development index [10]. High level of income at the country level (macro-level) can be associated with a better health system, hence better health outcomes at the individual level (micro-level) [26]. A high level of education can promote better knowledge of the mode of transmission and thus accurate preventive measures against these infections. However, it should be noted that we found an association with level of knowledge both for women and men. Again, this highlights that women are not the sole pivot of their own health, which emphasizes their dependency to the level of education of their male partner.

Therefore, we should think of a way to put women alone and as pivot in decision-making for their health, and how to have the contribution of men, without influence of men on women’s health. Women have better life-expectancy than men, but a higher disadvantage concerning morbidity. This is described as the female-male health-survival paradox [7]. Beyond biological considerations already well defined and recognized [2, 17], we should also take into account the influence of gender difference and development to address the burden of these infections in the continent. This is supported by the source of heterogeneity found for HCV prevalence. By contrasts, the prevalence of HCV infection increased only with decreasing proportion of seats held by women in the parliament. All these results emphasize the importance of the “women’s empowerment” concept in order to become the sole decision-maker of their own health and to ensure it.

The final model explained more than half of the heterogeneity of HBV prevalence while it explained less than 1% for HCV prevalence. This finding highlights the difference of the influence of gender-related human development indicators on the prevalence of these infections. The prevalence of HBV seems more dependent on grouping human development indicators than HCV. Unfortunately, because of missing data on individual risk factors in the various included studies, we were unable to include them in the multivariable meta-regression models. It is possible, and it remains to be investigated, that HCV prevalence in pregnant women would depend more on individual factors. Is this because HBV is transmitted both sexually and through the bloodstream while HCV is more dependent on the bloodstream [27]? Indeed, sexual behaviours in a group of individuals could depend on the fact that it belongs to this group. This would be consistent with holistic paradigm or macro-sociological approach: society encompasses individuals, and individual behaviour is seen only as a fragment of collective behaviour [28].

Notwithstanding, findings of this study should be interpreted with caution and in the context of its limitations. Firstly, we found substantial heterogeneity in estimating our prevalence estimates. Although we identified some sources of heterogeneity, there may still be others not investigated including participants’ characteristics of included studies. However, we were unable to assess these factors because they were not fully reported in primary data. Indeed, individuals’ characteristics were reported only between 3 and 66% in included studies. Secondly, countries and UNSD African regions were not uniformly represented and were not homogeneous in their grouping. This can limit the generalizability of findings to the entire continent. Thirdly, we found publication bias in some main analyses, suggesting that studies with low sample sizes could have altered the study findings. This study is, to the best of our knowledge, among the systematic review including meta-analysis of studies on the prevalence of HBV and HCV infections among pregnant women in Africa. Singularly, we investigated the association between prevalence estimates of hepatitis B and C virus infections and gender-related human development indicators. Strengths also include a comprehensive search strategy and involvement of pairs of independent investigators at all stages of the review process. Almost 82.8% of studies were assessed as having a low risk of bias in their methodological quality, suggesting that we can be confident in the quality of the findings. In addition, the sensitivity analysis including only studies with low risk yielded a very close prevalence to that estimated in the crude analysis. A multivariable meta-regression analysis was conducted helping to control potential confounders of source of prevalence variation, even if there remained some residual unexplained heterogeneity.

## Conclusions

This research depicted a high prevalence of HBV and HCV infections among pregnant women living in Africa with unequal distribution between UNSD regions and areas for HBV. HBV and HCV prevalence were 6.8 and 3.4%, respectively. The prevalence of HBV was higher in Central and Western Africa and in rural areas whereas there was no difference for HCV prevalence. Children born from an HBV or HCV positive mother were at high risk of getting infected since one out of five pregnant women and two out of three pregnant women can transmit HBV and HCV to their child, respectively. The prevalence of HBV infection significantly increased with some gender-related human development indicators including decreasing gender development index, decreasing males’ level of education, decreasing females’ expected years of schooling, and increasing gender inequality index. The prevalence of HCV infection increased with decreasing proportion of seats held by women in parliament. Therefore, to address the burden of these infections among pregnant women living in Africa, one should not only take into account the risk factors at the individual level already known but also macro-level factors including gender-related human development indicators and dwelling in rural areas.

## Additional files

Additional file 1: Multilingual abstracts in the five official working languages of the United Nations. (PDF 293 kb)

Additional file 2: Supplementary Tables and Figures. (PDF 4252 kb)

## Abbreviations

HBeAg: HBe antigen
HBsAg: HBs antigen
HBV: Hepatitis B virus
HCV: Hepatitis C virus
HCVAb: Antibodies against HCV
UNSD: United Nations Statistics Division
WHO: World Health Organization

## References

[1] World Health Organization. Global Health sector strategy on viral hepatitis 2016-2021: Towards ending viral hepatitis. http://apps.who.int/iris/bitstream/10665/246177/1/WHO-HIV-2016.06-eng.pdf?ua=1. Accessed 2 Feb 2018.

[2] World Health Organization. Global hepatitis report. http://www.who.int/hepatitis/publications/global-hepatitis-report2017/en/. Accessed 18 Feb 2018.

[3] Reddick KL, Jhaveri R, Gandhi M, James AH, Swamy GK (2011). Pregnancy outcomes associated with viral hepatitis. J Viral Hepat.

[4] Dunkelberg JC, Berkley EM, Thiel KW, Leslie KK (2014). Hepatitis B and C in pregnancy: a review and recommendations for care. J Perinatol.

[5] Mora N, Adams WH, Kliethermes S, Dugas L, Balasubramanian N, Sandhu J, Nde H, Small C, Jose J, Scaglione S, Layden JE (2016). A synthesis of Hepatitis C prevalence estimates in sub-Saharan Africa: 2000-2013. BMC Infect Dis.

[6] Ott JJ, Stevens GA, Wiersma ST (2012). The risk of perinatal hepatitis B virus transmission: hepatitis B e antigen (HBeAg) prevalence estimates for all world regions. BMC Infect Dis.

[7] Mayor E (2015). Gender roles and traits in stress and health. Front Psychol.

[8] Wolk DM, Jones MF, Rosenblatt JE (2001). Laboratory diagnosis of viral hepatitis. Infect Dis Clin N Am.

[9] United Nations Statistics Division. Standard country or area codes for statistical use (M49). https://unstats.un.org/unsd/methodology/m49/. Accessed 15 Jan 2018.

[10] United Nations Development Programme. International Human Development Indicators. http://hdr.undp.org/en/countries. Accessed 15 Jan 2018.

[11] Hoy D, Brooks P, Woolf A, Blyth F, March L, Bain C, Baker P, Smith E, Buchbinder R (2012). Assessing risk of bias in prevalence studies: modification of an existing tool and evidence of interrater agreement. J Clin Epidemiol.

[12] Barendregt JJ, Doi SA, Lee YY, Norman RE, Vos T (2013). Meta-analysis of prevalence. J Epidemiol Community Health.

[13] Higgins JP, Thompson SG (2002). Quantifying heterogeneity in a meta-analysis. Stat Med.

[14] Cochran WG (1954). The combination of estimates from different experiments. Biometrics..

[15] Egger M, Davey Smith G, Schneider M, Minder C (1997). Bias in meta-analysis detected by a simple, graphical test. BMJ.

[16] Viera AJ, Garrett JM (2005). Understanding interobserver agreement: the kappa statistic. Fam Med.

[17] Gasim GI, Murad IA, Adam I (2013). Hepatitis B and C virus infections among pregnant women in Arab and African countries. J Infect Dev Ctries.

[18] Madhava V, Burgess C, Drucker E (2002). Epidemiology of chronic hepatitis C virus infection in sub-Saharan Africa. Lancet Infect Dis.

[19] UNAIDS. 90–90–90 - An ambitious treatment target to help end the AIDS epidemic. http://www.unaids.org/en/resources/documents/2017/90-90-90. Accessed 25 May 2018.

[20] Bigna JJ, Amougou MA, Asangbeh SL, Kenne AM, Nansseu JR (2017). Seroprevalence of hepatitis C virus infection in Cameroon: a systematic review and meta-analysis. BMJ Open.

[21] Bigna JJ, Amougou MA, Asangbeh SL, Kenne AM, Noumegni SRN, Ngo-Malabo ET, Noubiap JJ (2017). Seroprevalence of hepatitis B virus infection in Cameroon: a systematic review and meta-analysis. BMJ Open.

[22] Nelson NP, Jamieson DJ, Murphy TV (2014). Prevention of perinatal Hepatitis B virus transmission. J Pediatr Infect Dis Soc.

[23] Spearman CW, Afihene M, Ally R, Apica B, Awuku Y, Cunha L, Dusheiko G, Gogela N, Kassianides C, Kew M (2017). Hepatitis B in sub-Saharan Africa: strategies to achieve the 2030 elimination targets. Lancet Gastroenterol Hepatol.

[24] Lee C, Gong Y, Brok J, Boxall EH, Gluud C. Effect of hepatitis B immunisation in newborn infants of mothers positive for hepatitis B surface antigen: systematic review and meta-analysis. BMJ (Clinical research ed). 2006;332(7537):328-36. 10.1136/bmj.38719.435833.7C PMC1363909 16443611

[25] World Health Organization. WHO-UNICEF estimates of HepB_BD coverage. http://apps.who.int/immunization_monitoring/globalsummary/timeseries/tswucoveragehepb_bd.html. Accessed 2 Feb 2018.

[26] Baker DP, Leon J, Smith Greenaway EG, Collins J, Movit M (2011). The education effect on population health: a reassessment. Popul Dev Rev.

[27] Thursz M, Fontanet A (2014). HCV transmission in industrialized countries and resource-constrained areas. Nat Rev Gastroenterol Hepatol.

[28] Grosseti M. L'espace à trois dimensions des phénomènes sociaux. http://journals.openedition.org/sociologies/3466. Accessed 22 Feb 2018.

