# Supplementary material for: Gender development and hepatitis B and C infections among pregnant women in Africa: a systematic review and meta-analysis
Source: Infect Dis Poverty. 2019 Mar 4;8:16. doi: 10.1186/s40249-019-0526-8 (PMC6398223; doi:10.1186/s40249-019-0526-8)
Supplement: Supplementary file 1 — Multilingual abstracts in the five official working languages of the United Nations. (PDF 293 kb) [file 40249_2019_526_MOESM1_ESM.pdf]

Translation of the abstract into the five official working languages of the United Nations

العلاقة بين مؤشرات التنمية البشرية حسب نوع الجنس وفيروس التهاب الكبد الوبائي بي و سي لدى النساء الحوامل في أفريقيا: مراجعة منهجية وتحليل تجميعي

جان جويل بيغنا ، أنجيلادين م. كين ، آغايلز هامرون ، ماري س. ندانغانغ ، أودري جويس فوكا ، داليا نويل تونوغا ، ريمي ليناين ، ماري أ. أموغو ، جوبرت ريتشي نانسو

الملخّص
الخلفية: في حين أنَّ أفريقيا منطقة استيطان لفيروسيّ الالتهاب الكبدى الوبائى بى (HBV) و سى (HCV)، هناك محدودية في المعلومات المتوفرة حول المشاكل التي يسببها هذان الفيروسان لدى النساء الحوامل بها. تهدف هذه المراجعة المنهجية والتحليل التجميعي إلى تحديد حجم انتشار هذه العدوى بين النساء الحوامل اللواتي يعشن في أفريقيا والتاكد من مدى ارتباطها بمؤشرات التنمية البشرية حسب نوع الجنس..
النص الأساسي: استعنّا في البحث بمحرك PubMed وقاعدة البيانات Embase وشبكة العلوم وموقع منظمة أفريكان جورنال أونلاين والمؤشر الطبي العالمي Global Index Medicus، دون التقيد بلغة معينة، لمعرفة الدراسات القائمة على الملاحظة حول الإصابات بفيروس الالتهاب الكبدى الوبائى بى و سى لدى النساء الحوامل المقيمات في أفريقيا المنشورة في الفترة من 1 يناير 2000 حتى 31 ديسمبر 2017 . بينت الدراسات المطابقة لمعايير البحث معدل انتشار عدوى فيروس التهاب الكبد الوبائي بي أو فيروس الالتهاب الكبدي سي (أو كليهما) (المستضد السطحي لالتهاب الكبدي بي والأجسام المضادة لفيروس الالتهاب الكبدي سي) والقدرة على العدوى (المستضد إي أو الحمل الفيروسي لفيروس الالتهاب الكبدي سي القابل للكشف) (أو كليهما)، مع مراجعة كل دراسة على حدة للحصول على جودة منهجية. استخدمنا نموذج التأثيرات العشوائية للتحليل التجميعي في تجميع الدراسات. و تم تضمين إجمالى 145 دراسة (258, 251 مشارك من 30 دولة) و تم تقسيمهم كالتالي: 120 (82.8%) لديهم ميل منخفض للعدوى و 24 (16.5%) لديهم ميل متوسط و حالة واحدة (0.7%) لديها نسبة خطورة عالية. كان معدل انتشار عدوى فيروس التهاب الكبد الوبائي بي وسيبنسبة 6.8٪ %(مجال الثقة (95%): [*CI*] 6.1-7.6 لعدد 113 دراسة و 3.4%(مجال الثقة (95%): 2.6-4.2 لعدد 58 دراسة). كان معدل انتشار المستضد إي والحمل الفيروسي لفيروس الالتهاب الكبدي سي القابل للكشف 18.9 ٪ (مجال الثقة 95 ٪: 14.4 -23.9) و 62.3 ٪ (مجال الثقة 95 ٪: 51.6–72.5) لدى النساء الحوامل المصابات بفيروسيّ الالتهاب الكبدي بي وسي. أظهر تحليل التحوف التلوي متعدد المتغيرات أن انتشار العدوى بفيروس الالتهاب الكبدي الوبائي بي ازداد مع انخفاض مؤشر التنمية حسب نوع الجنس ومستوى تعليم الذكور وعدد سنوات الدراسة المتوقعة من الإناث. علاوة على ذلك، كان هذا الانتشار أعلى في المناطق الريفية وفي غرب أفريقيا ووسطها. زاد معدل انتشار عدوى فيروس التهاب الكبد الوبائي سي مع تناقص نسبة المقاعد التي تشغلها النساء في البرلمان.
الاستنتاجات: لمعالجة عبء العدوى بفيروس التهاب الكبد الوبائي بي و سي، وإلى جانب عوامل الخطر المعروفة على المستوى الفردي، ينبغي مراعاة العوامل على المستوى الكلي، بما في ذلك مؤشرات التنمية البشرية حسب نوع الجنس والسكن في المناطق الريفية. ففي أفريقيا، يبدو أنَّ هناك احتمالية كبيرة بأنَّ تنقل الأمهات المصابات بفيروسيّ التهاب الكبدي الوبائي بي وسي العدوى لأطفالهم.

Translated from English version into Arabic by Rana Chehab and Heba Kandel, through

TRANSLATORS
WITHOUT BORDERS

非洲孕妇的性别角色发展与乙型和丙型肝炎感染：系统综述和荟萃分析

Jean Joel Bigna, Angeladine M. Kenne, Aghiles Hamroun, Marie S. Ndangang, Audrey Joyce Foka, Dahlia Noelle Tounouga, Rémi Lenain, Marie A. Amougou, Jobert Richie Nansseu

摘要

引言：在非洲，尽管乙型肝炎(HBV)和丙型肝炎(HCV)的感染较高，孕妇的疾病负担数据却缺失。本系统综述和荟萃分析旨在确定非洲孕妇中 HBV 和 HCV 感染的规模，并分析其与与性别有关的人类发展指标的关系。

正文：在不受语言限制的前提下，我们检索 PubMed、Embase、Web of Science、Africa Journal Online 和 Global Index Medicus，以确定 2000 年 1 月 1 日至 2017 年 12 月 31 日期间发表的关于非洲孕妇 HBV 和 HCV 感染的观察性研究。这些研究报告了 HBV 和/或 HCV 感染(HBs 抗原和 HCV 抗体)和/或感染性(HBe 抗原或可检测到的 HCV 病毒载量)的流行情况。我们对每项研究都独立地进行了方法学质量审查，并使用随机效应模型的荟萃分析来对研究进行汇总。共纳入 145 项研究(258 251 名参与者，30 个国家)，其中 120 项(82.8%)为低分组，24 项(16.5%)为中分组，1 项(0.7%)为高分组。HBV 和 HCV 感染的流行率分别为 6.8%(95%置信区间[*CI*]: 6.1–7.6, 113 项研究)和 3.4% (95% *CI*: 2.6–4.2, 58 项研究)。HBe 抗原和 HCV 检测病毒载量在 HBV 阳性和 HCV 阳性孕妇中分别为 18.9% (95% *CI*: 14.4–23.9)和 62.3% (95% *CI*: 51.6–72.5)。多元回归分析显示，随着性别角色发展指数、男性受教育水平和女性受教育年限的降低，乙肝病毒感染的流行程度增加。此外，这种流行趋势在农村地区和西非和中非更为常见。随着妇女在议会中所占席位比例的减少，丙肝病毒感染的流行程度加重。

结论：为了解决乙肝病毒和丙肝病毒感染的负担，除了众所周知的个人一级危险因素外，还应考虑包括与性别有关的人类发展指标和农村地区居住的宏观因素。在非洲，感染 HBV 或 HCV 的母亲极有可能将病毒传播给其子女。

Translated from English version into Chinese by Cong-Shan Liu, edited by Jin Chen

Développement dans l'égalité des sexes et hépatites B et C chez les femmes enceintes en Afrique : revue systématique et méta-analyse

Jean Joël Bigna, Angeladine M. Kenne, Aghiles Hamroun, Marie S. Ndangangang, Audrey Joyce Foka, Dahlia Noëlle Tounouga, Rémi Lenain, Marie A. Amougou et Jobert Richie Nansseu

Résumé

Contexte : Bien que l'hépatite virale B (VHB) et l'hépatite virale C (VHC) soient hyperendémiques en Afrique, on dispose de peu de données sur le fardeau que ces maladies font peser sur les femmes enceintes. La revue systématique et la méta-analyse présentées ici visent à déterminer l'ampleur de ces infections chez les femmes enceintes vivant en Afrique et à étudier leur association avec les indicateurs sexospécifiques du développement.

Corps du texte : Nous avons effectué des recherches dans PubMed, Embase, Web of Science, Africa Journal Online et Global Index Medicus, sans restriction de langue, pour trouver des études

observationnelles sur les infections à VHB et à VHC chez les femmes enceintes résidant en Afrique, publiées entre le 1er janvier 2000 et le 31 décembre 2017. Les études pertinentes font état de la prévalence de l'infection par le VHB et/ou le VHC (antigène HBs et anticorps anti-VHC) et/ou de l'infectiosité (antigène HBe ou charge virale détectable du VHC). La qualité méthodologique de chacune de ces études a été vérifiée indépendamment. Nous avons utilisé un modèle de méta-analyse à effets aléatoires pour regrouper les études. Au total, 145 études (258 251 participantes dans 30 pays) ont été incluses, dont 120 (82,8 %) présentaient un risque faible de biais, 24 (16,5 %) un risque modéré et une (0,7 %) un risque élevé. La prévalence des infections par le VHB et le VHC était de 6,8 % (intervalle de confiance [*IC*] à 95 % de 6,1) 7,6, 113 études) et 3,4 % (*IC* à 95 % de 2,6 à 4,2, 58 études), respectivement. La prévalence de l'antigène HBe et de la charge virale de VHC décelable était de 18,9 % (*IC* à 9 % de 14,4 à 23,9) et 62,3 % (*IC* à 95 % de 51,6 à 72,5) chez les femmes enceintes séropositives au VHB et au VHC, respectivement. La méta-analyse de régression multivariée a montré que la prévalence de l'infection par le VHB était inversement proportionnelle aux indicateurs sexospécifiques du développement, au niveau d'éducation des hommes et aux années de scolarité attendues pour les femmes. Qui plus est, cette prévalence était plus élevée dans les zones rurales et en Afrique occidentale et centrale. La prévalence de l'infection par le VHC était inversement proportionnelle à la proportion de sièges occupés par des femmes au parlement.

Conclusions : Pour s'attaquer au fardeau des infections à VHB et à VHC, au-delà des facteurs de risque individuel bien connus, il convient de tenir compte des facteurs macroéconomiques, notamment des indicateurs sexospécifiques de développement et des conditions de logement dans les zones rurales. En Afrique, il semble exister un potentiel élevé de transmission du virus par les mères infectées par le VHB ou le VHC à leurs enfants.

Translated from English version into French by Chantal Quintric Leveille and Suzanne Assenat, through

Гендерное развитие и заражение вирусом гепатитов В и С среди беременных женщин в Африке: систематический обзор и метаанализ

Жан Жоель Бинья (Jean Joel Bigna), Ангеладин М. Кенне (Angeladine M. Kenne), Агилас Хамрун (Aghiles Hamroun), Мари С. Нданганг (Marie S. Ndangang), Одри Джойс Фока (Audrey Joyce Foka), Далия Ноель Тунуга (Dahlia Noelle Tounouga), Реми Ленен (Rémi Lenain), Мари А. Амугу (Marie A. Amougou), Жобер Ричи Нансо (Jobert Richie Nansseu)

Аннотация

Вводная информация: Хотя Африка является чрезвычайно эндемичным регионом для вирусных гепатитов В (HBV) и С (HCV), существует мало информации об соответствующей заболеваемости среди беременных женщин. Данный систематический обзор и метаанализ призван определить масштаб этих инфекций среди беременных женщин, проживающих в

Африке, и изучить их взаимосвязь с показателями развития человеческого потенциала с учётом гендерного фактора.

Основная часть: Мы просмотрели базы PubMed, Embase, Web of Science, Africa Journal Online и Global Index Medicus, без ограничений по языку, чтобы найти наблюдательные исследования об инфицировании вирусом гепатита В и С у беременных женщин, проживающих в Африке, опубликованные в период с 1 января 2000 года до 31 декабря 2017 года. Согласно исследованиям, подходящим под критерии, распространенность инфекций вирусов гепатита В и С (антигены вируса гепатита В и антитела к вирусу гепатита С) и/или инфекционности (HBe-антиген или обнаруживаемое наличие вируса гепатита В). Каждое исследование независимо проверялось на методологическую достоверность. Мы выбрали модель со случайными эффектами для метаанализа совокупности исследований. В целом, в список были включены 145 исследований (258 251 участник из 30 стран), из которых 120 (82,8%) имели низкий, 24 (16,5%) средний, and одно (0,7%) высокий риск систематической погрешности. Распространенность заражения вирусом гепатита В и С составила6,8% (доверительный интервал 95% [*ДИ*]: 6.1–7.6, 113 исследований) и 3,4% (95% *ДИ*: 2.6–4.2, 58 исследований), соответственно. Распространенность HBe-антигена вируса гепатита В или явного наличия вируса гепатита С составляли 18,9% (95% *ДИ*: 14.4–23.9) и 62,3% (95% *ДИ*: 51.6–72.5) среди инфицированных вирусом гепатита В и гепатита С беременных женщин, соответственно. Многомерный анализ методом метарегрессии показал, что распространенность заражения вирусом гепатита В возрастала вместе с уменьшением показателя гендерного развития, уровня образования среди мужчин и ожидаемых лет обучения среди женщин. Более того, эта распространенность была несколько выше в сельских районах и в западной и центральной Африке. Распространенность заражения вирусом гепатита С возрастала наряду с уменьшением доли женщин в парламенте.

Выводы: Анализируя заболеваемость вирусом гепатита В и С, помимо известных факторов риска на индивидуальном уровне, следует учитывать и факторы на макроуровне, такие как индекс человеческого развития с учётом гендерных факторов и проживание в сельской местности. В Африке матери, зараженные вирусом гепатитов В и С, похоже, имеют высокий риск передачи болезни своим детям.

Translated from English version into Russian by Malika El Khadhri and Amro Salah, through

TRANSLATORS WITHOUT BORDERS

Développement dans l'égalité des sexes et hépatites B et C chez les femmes enceintes en Afrique : revue systématique et méta-analyse

Jean Joel Bigna, Angeladine M. Kenne, Aghiles Hamroun, Marie S. Ndangang, Audrey Joyce Foka, Dahlia Noelle Tounouga, Rémi Lenain, Marie A. Amougou, Jobert Richie Nansseu

Resumen

Antecedentes: si bien África es una región hiperendémica de infecciones por el virus de la hepatitis B (VHB) y C (VHC), hay pocos datos de la carga consiguiente en mujeres embarazadas. Esta revisión sistemática y metaanálisis tiene como objetivo determinar la magnitud de estas infecciones entre las mujeres embarazadas que viven en África y también investigar su asociación con los indicadores de desarrollo humano relacionados con el género.

Texto principal: hicimos una búsqueda en PubMed, Embase, Web of Science, Africa Journal Online y Global Index Medicus, sin restricciones de idioma, para identificar estudios observacionales sobre las infecciones por el VHB y el VHC en mujeres embarazadas que viven en África y publicados desde el 1 de enero de 2000 hasta el 31 de diciembre de 2017. Los estudios elegibles informaron la prevalencia de las infecciones por el VHB y/o el VHC (antígeno HBs y anticuerpos VHC) y/o la infectividad (antígeno HBe o carga viral detectable de VHC). La calidad metodológica de cada uno de los estudios fue evaluada de forma independiente. Utilizamos un metaanálisis con un modelo de efectos aleatorios para combinar los estudios. En total, se incluyeron 145 estudios (258.251 participantes en 30 países) de los cuales 120 (82,8%) presentaron un sesgo bajo, 24 (16,5%) un sesgo moderado y un estudio (0,7%) tuvo un alto riesgo de sesgo. La prevalencia de las infecciones por el VHB y el VHC fue del 6,8% (95% de intervalo de confianza [*IC*]: 6,1–7,6; 113 estudios) y 3,4% (95% *IC*: 2,6–4,2; 58 estudios), respectivamente. La prevalencia del antígeno HBe y la carga viral detectable fue 18,9% (95% *IC*: 14,4–23,9) y 62,3% (95% *IC*: 51,6–72,5) en mujeres embarazadas VHB y VHC positivas, respectivamente. El análisis multivariable de meta regresión mostró que la prevalencia de la infección por el VHB aumentó al disminuir el índice de desarrollo por género, el nivel educativo de los hombres y la escolaridad esperada de las mujeres. Además, esta prevalencia fue mayor en las zonas rurales y en el África occidental y central. La prevalencia de la infección por el VHC aumentó en la medida que disminuía la cantidad de bancas ocupadas por mujeres en el parlamento.

Conclusiones: a fin de abordar la carga de las infecciones por el VHB y el VHC, más allá de los factores de riesgo conocidos a nivel individual, entre los factores a nivel macro debemos considerar los indicadores de desarrollo humano relacionados con el género y la vivienda en zonas rurales. En África, las madres infectadas por el VHB o el VHC parecen tener un elevado potencial de transmisión a sus hijos.

Translated from English version into Spanish by Diana Kreimer and Ivana Benzaquen, through

TRANSLATORS
WITHOUT BORDERS
