# Supplementary material for: Gender development and hepatitis B and C infections among pregnant women in Africa: a systematic review and meta-analysis
Source: Infect Dis Poverty. 2019 Mar 4;8:16. doi: 10.1186/s40249-019-0526-8 (PMC6398223; doi:10.1186/s40249-019-0526-8)

Hepatitis B and C infections prevalence in pregnant women and association with gender related human development indicators in Africa

a systematic review and meta-analysis

Jean Joel **Bigna**, Angeladine M. **Kenne**, Aghiles **Hamroun**,

Marie S. **Ndangang**, Audrey Joyce **Foka**, Dahlia Noelle **Tounouga**,

Rémi **Lenain**, Marie A. **Amougou**, Jobert Richie **Nansseu**

Appendix

Supplemental Table 1 : Search strategy in Embase 2
Supplemental Table 2 : Reference list of included studies 3
Supplemental Table 3 : List of countries 10
Supplemental Table 4 : Individual characteristics of included studies 11
Supplemental Table 5 : Sources of heterogeneity of the prevalence of HBV infection in pregnant women in Africa 21
Supplemental Table 6 : Sources of heterogeneity of the prevalence of HCV infection in pregnant women in Africa 22

Supplemental Figure 1 : Meta-analysis results for HBV infection prevalence among pregnant women in Africa, by country 23
Supplemental Figure 2 : Meta-analysis results for HCV infection prevalence among pregnant women in Africa, by country 24
Supplemental Figure 3 : Funnel plot for HBV infection prevalence among pregnant women in Africa 25
Supplemental Figure 4 : Funnel plot for HCV infection prevalence among pregnant women in Africa 26
Supplemental Figure 5 : Meta-analysis results for HBV infection prevalence among pregnant women in Africa, by area 27
Supplemental Figure 6 : Meta-analysis results for HCV infection prevalence among pregnant women in Africa, by area 28

Supplemental Table 1 : ***Search strategy in Embase***

**Search**	**Query**
#6	#1 AND #2 AND #3 AND #4 AND #5
#5	'hepatitis b'/exp OR 'hepatitis b' OR 'viral hepatitis b' OR 'hepatitis b virus'/exp OR 'hepatitis b virus' OR 'hbv'/exp OR 'hbv' OR 'hepatitis c'/exp OR 'hepatitis c' OR 'viral hepatitis c' OR 'hepatitis c virus'/exp OR 'hepatitis c virus' OR 'hcv'
#4	'article'/it OR 'article in press'/it OR 'letter'/it OR 'short survey'/it
#3	[2000-2017]/py
#2	africa* OR 'algeria'/exp OR algeria OR 'angola'/exp OR angola OR 'benin'/exp OR benin OR 'botswana'/exp OR botswana OR 'burkina faso'/exp OR 'burkina faso' OR 'burundi'/exp OR burundi OR 'cameroon'/exp OR cameroon OR 'canary islands'/exp OR 'canary islands' OR 'cape verde'/exp OR 'cape verde' OR 'central african republic'/exp OR 'central african republic' OR 'chad'/exp OR chad OR 'comoros'/exp OR comoros OR 'congo'/exp OR congo OR 'democratic republic of congo' OR 'djibouti'/exp OR djibouti OR 'egypt'/exp OR egypt OR 'equatorial guinea'/exp OR 'equatorial guinea' OR 'eritrea'/exp OR eritrea OR 'ethiopia'/exp OR ethiopia OR 'gabon'/exp OR gabon OR 'gambia'/exp OR gambia OR 'ghana'/exp OR ghanaOR 'guinea'/exp OR guinea OR 'guinea bissau'/exp OR 'guinea bissau' OR 'ivory coast'/exp OR 'ivory coast' OR 'cote ivoire' OR jamahiriya OR 'kenya'/exp OR kenya OR 'lesotho'/exp OR lesotho OR 'liberia'/exp OR liberia OR 'libya'/exp OR libya OR 'madagascar'/exp OR madagascar OR 'malawi'/exp OR malawi OR 'mali'/exp OR mali OR 'mauritania'/exp OR mauritania OR 'mauritius'/exp OR mauritius OR 'mayotte'/exp OR mayotteOR 'morocco'/exp OR morocco OR 'mozambique'/exp OR mozambique OR 'namibia'/exp OR namibia OR 'niger'/exp OR niger OR 'nigeria'/exp OR nigeria OR principe OR 'reunion'/exp OR reunion OR 'rwanda'/exp OR rwanda OR 'sao tome' OR 'senegal'/exp OR senegal OR 'seychelles'/exp OR seychelles OR 'sierra leone'/exp OR 'sierra leone' OR 'somalia'/exp OR somalia OR 'south africa'/exp OR 'south africa' OR 'st helena'/exp OR 'st helena' OR 'sudan'/exp OR sudan OR 'swaziland'/exp OR swaziland OR 'tanzania'/exp OR tanzania OR 'togo'/exp OR togo OR 'tunisia'/exp OR tunisia OR 'uganda'/exp OR uganda OR 'western sahara'/exp OR 'western sahara' OR 'zaire'/exp OR zaire OR 'zambia'/exp OR zambia OR 'zimbabwe'/exp OR zimbabwe OR 'central africa'/exp OR 'central africa' OR 'central african'/exp OR 'central african' OR 'west africa'/exp OR 'west africa' OR 'west african'/exp OR 'west african' OR 'western africa'/exp OR 'western africa' OR 'western african'/exp OR 'western african' OR 'east africa'/exp OR 'east africa' OR 'east african'/exp OR 'east african' OR 'eastern africa'/exp OR 'eastern africa' OR 'eastern african'/exp OR 'eastern african' OR 'north africa'/exp OR 'north africa' OR 'north african'/exp OR 'north african' OR 'northern africa'/exp OR 'northern africa' OR 'northern african'/exp OR 'northern african' OR 'south african'/exp OR 'south african' OR 'southern africa'/exp OR 'southern africa' OR 'southern african'/exp OR 'southern african' OR 'sub saharan africa'/exp OR 'sub saharan africa' OR 'sub saharan african' OR 'subsaharan africa'/exp OR 'subsaharan africa'
#1	'pregnant women'/exp OR 'pregnant women' OR 'pregnancy'/exp OR 'pregnancy' OR 'pregnant' OR 'prenatal'/exp OR 'prenatal' OR 'prenatal care'/exp OR 'prenatal care' OR 'gestation'/exp OR 'gestation'

Supplemental Table 2 : **Reference list of included studies**

1.	Aba HO, Aminu M. Seroprevalence of hepatitis B virus serological markers among pregnant Nigerian women. *Ann Afr Med* 2016; 15(1):20-7.
2.	AbdulQawi K, Youssef A, Metwally MA, Ragih I, AbdulHamid M, Shaheen A. Prospective study of prevalence and risk factors for hepatitis C in pregnant Egyptian women and its transmission to their infants. *Croatian medical journal* 2010; 51(3): 219-28.
3.	Adabara NU, Ajala OO, Momohjimoh A, Hashimu Z, Agabi AYV. Prevalence of hepatitis B virus among women attending antenatal clinic in the general hospital, Minna, Niger State. *Shiraz E Medical Journal* 2012; 13(1): 28-32.
4.	Adegbesan-Omilabu MA, Okunade KS, Gbadegesin A, Olowoselu OF, Oluwole AA, Omilabu SA. Seroprevalence of hepatitis B virus infection among pregnant women at the antenatal booking clinic of a Tertiary Hospital in Lagos Nigeria. *Nigerian journal of clinical practice* 2015; 18(6): 819-23.
5.	Adesina O, Oladokun A, Akinyemi O, et al. Human immuno-deficiency virus and hepatitis B virus coinfection in pregnancy at the University College Hospital, Ibadan. *African journal of medicine and medical sciences* 2010; 39(4): 305-10.
6.	Agbenu E, Banla A, Kolou M, et al. Serological markers used for hepatitis B surveillance in Togo: Status report and action proposals. *Med Trop* 2008; 68(6): 621-4.
7.	Aidaoui M, Bouzbid S, Laouar M. Seroprevalence of HIV infection in pregnant women in the Annaba region (Algeria). *Revue d'Epidemiologie et de Sante Publique* 2008; 56(4): 261-6.
8.	Ajugwo AO, Nwoke BEB, Ozims SJ, Eberendu IF, Nwibana BK. Prevalence 0f Hbsag and Haematological Parameters Among Pregnant Women Attending a Nigerian Tertiary Hospital. *Acta Scientific Nutritional Health* 2017; 1(3):55-60.
9.	Alegbeleye JO, Nyengidiki TK, Ikimalo JI. Maternal and neonatal seroprevalence of hepatitis B surface antigen in a hospital based population in South-South, Nigeria. *International Journal of Medicine and Medical Sciences* 2013; 5(5):241-46.
10.	Aluor EPT, Oluma HOA, Ega RAI, Owoicho N. SERO-epidemiological survey and risk factors for Hepatitis B virus (HBV) infection among pregnant women in Logo LGA, Benue State, Nigeria. *African Journal of Clinical and Experimental Microbiology* 2016; 17(1): 66-75.
11.	Anaedobe CG, Fowotade A, Omoruyi CE, Bakare RA. Prevalence, socio-demographic features and risk factors of Hepatitis B virus infection among pregnant women in Southwestern Nigeria. *Pan African Medical Journal* 2015; 20:406.
12.	Andersson MI, Maponga TG, Ijaz S, et al. The epidemiology of hepatitis B virus infection in HIV-infected and HIV-uninfected pregnant women in the Western Cape, South Africa. *Vaccine* 2013; 31(47): 5579-84.
13.	Andersson MI, Maponga TG, Ijaz S, Theron G, Preiser W, Tedder RS. High HBV viral loads in HIV-infected pregnant women at a tertiary hospital, South Africa. *Journal of Acquired Immune Deficiency Syndromes* 2012; 60(4): e111-e2.
14.	Andreotti M, Pirillo MF, Liotta G, et al. The impact of HBV or HCV infection in a cohort of HIV-infected pregnant women receiving a nevirapine-based antiretroviral regimen in Malawi. *BMC Infectious Diseases* 2014; 14(1).
15.	Apea-Kubi KA, Yamaguchi S, Sakyi B, Ofori-Adjei D. HTLV-1 and other viral sexually transmitted infections in antenatal and gynaecological patients in Ghana. *West African Journal of Medicine* 2006; 25(1): 17-21.
16.	Araya Mezgebo T, Niguse S, Gebrekidan Kahsay A, Hailekiros H, Berhe N, Asmelash Dejene T. Hepatitis B virus infection and associated risk factors among pregnant women attending antenatal care in health facilities of Tigray, Northern Ethiopia. *Journal of Medical Virology* 2017 ; 90(3) :503-509.
17.	Bayo P, Ochola E, Oleo C, Mwaka AD. High prevalence of hepatitis B virus infection among pregnant women attending antenatal care: A cross-sectional study in two hospitals in northern Uganda. *BMJ Open* 2014; 4(11) : e005889.
18.	Burnett RJ, Ngobeni JM, François G, et al. Increased exposure to hepatitis B virus infection in HIV-positive South African antenatal women. *International Journal of STD and AIDS* 2007; 18(3): 152-6.
19.	Candotti D, Danso K, Allain JP. Maternofetal transmission of hepatitis B virus genotype E in Ghana, west Africa. *Journal of General Virology* 2007; 88(10): 2686-95.
20.	Chasela CS, Kourtis AP, Wall P, et al. Hepatitis B virus infection among HIV-infected pregnant women in Malawi and transmission to infants. *Journal of Hepatology* 2014; 60(3): 508-14.
21.	Chasela CS, Wall P, Drobeniuc J, et al. Prevalence of hepatitis C virus infection among human immunodeficiency virus-1-infected pregnant women in Malawi: The BAN study. *Journal of Clinical Virology* 2012; 54(4): 318-20.

22.	Cho Y, Bonsu G, Akoto-Ampaw A, et al. The prevalence and risk factors for hepatitis B surface Ag positivity in pregnant women in eastern region of Ghana. *Gut and Liver* 2012; 6(2): 235-40.
23.	Chotun N, Preiser W, Van Rensburg CJ, et al. Point-of-care screening for hepatitis B virus infection in pregnant women at an antenatal clinic: A South African experience. *PLoS ONE* 2017; 12(7) : e0181267.
24.	Collenberg E, Ouedraogo T, Ganamé J, et al. Seroprevalence of six different viruses among pregnant women and blood donors in rural and urban Burkina Faso: A comparative analysis. *Journal of Medical Virology* 2006; 78(5): 683-92.
25.	Daniels, AO, Ogundeyi, SB, Owanikin, SR. Sero-Prevalence of HBs Ag and HCV Antigenaemia Among Pregnant Women in Owo, South-West, Nigeria. *Glo Adv Res J Microbiol* 2017; 6(3): 8-12.
26.	De Paschale M, Ceriani C, Cerulli T, et al. Prevalence of HBV, HDV, HCV, and HIV Infection During Pregnancy in Northern Benin. *Journal of Medical Virology* 2014; 86:1281–1287.
27.	Desalegn Z, Wassie L, Beyene HB, Mihret A, Ebstie YA. Hepatitis B and human immunodeficiency virus co-infection among pregnant women in resource-limited high endemic setting, Addis Ababa, Ethiopia: Implications for prevention and control measures. *European Journal of Medical Research* 2016; 21 :16.
28.	Dionne-Odom J, Mbah R, Rembert NJ, et al. Hepatitis B, HIV, and Syphilis Seroprevalence in Pregnant Women and Blood Donors in Cameroon. *Infectious Diseases in Obstetrics and Gynecology* 2016; 2016 : 4359401
29.	Ducancelle A, Abgueguen P, Birguel J, et al. High endemicity and low molecular diversity of hepatitis B virus infections in pregnant women in a rural district of north cameroon. *PLoS ONE* 2013; 8(11) : e80346.
30.	Duru MU, Aluyi HS, Anukam KC. Rapid screening for co-infection of HIV and HCV in pregnant women in Benin City, Edo State, Nigeria. *Afr Health Sci* 2009; 9(3): 137-42.
31.	Eke AC, Eke UA, Okafor CI, Ezebialu IU, Ogbuagu C. Prevalence, correlates and pattern of hepatitis B surface antigen in a low resource setting. *Virology Journal* 2011; 8 : 12.
32.	El-Kamary SS, Hashem M, Saleh DA, et al. Reliability of risk-based screening for hepatitis C virus infection among pregnant women in Egypt. *Journal of Infection* 2015; 70(5): 512-9.
33.	El-Karaksy HM, Mohsen LM, Saleh DA, et al. Applicability and efficacy of a model for prevention of perinatal transmission of hepatitis B virus infection: single center study in Egypt. *World J Gastroenterol* 2014; 20(45): 17075-83.
34.	El-Magrahe H, Furarah AR, El-Figih K, El-Urshfany S, Ghenghesh KS. Maternal and neonatal seroprevalence of Hepatitis B surface antigen (HBsAg) in Tripoli, Libya. *Journal of Infection in Developing Countries* 2010; 4(3): 168-70.
35.	Elrazek A, Amer M, El-Hawary B, et al. Prediction of HCV vertical transmission: what factors should be optimized using data mining computational analysis. Liver International 2017; 37(4): 529-33.
36.	Elsheikh RM, Daak AA, Elsheikh MA, Karsany MS, Adam I. Hepatitis B virus and hepatitis C virus in pregnant Sudanese women. *Virology Journal* 2007; 4 : 104.
37.	Enow RT, Teyim P, Kamga HL, Neba ES, Nkuo-Akenji T. Sero-prevalence of Human Immunodeficiency Virus and hepatitis viruses and their correlation with CD4 T-cell lymphocyte counts in pregnant women in the Buea Health District of Cameroon. *International Journal of Biological and Chemical Sciences* 2016; 10(1):219-31.
38.	Ephraim R, Donko I, Sakyi SA, Ampong J, Agbodjakey H. Seroprevalence and risk factors of hepatitis B and hepatitis C infections among pregnant women in the asante akim north municipality of the ashanti region, Ghana; a cross sectional study. *African Health Sciences* 2015; 15(3): 709-13.
39.	Erhabor O, Onuigwe FU, Iwueke I, Bello L, Mohammad SY. Hepatitis B Surface Antigen Among Low-Risk Obstetric Population Attending Specialist Hospital Sokoto, North Western Nigeria. J Nurs Womens Health 2017; 5: JNWH127.
40.	Esan AJ, Omisakin CT, Ojo-Bola T, Owoseni MF, Fasakin KA, Ogunleye AA. Sero-Prevalence of Hepatitis B and Hepatitis C Virue Co-Infection among Pregnant Women in Nigeria. American Journal of Biomedical Research 2014; 2(1):11-15.
41.	Evelyn ME, Buseri FI, Wachukwu CK, Nnatuanya IN. Effects of hepatitis B infection on haemtological parameters in pregnancy in Port Harcourt, Nigeria. *Research Journal of Medical Sciences* 2009; 3(6): 194-7.
42.	Ezegbudo CN, Agbonlahor DE, Nwobu GO, et al. The seroprevalence of hepatitis B surface antigen and human immunodeficiency virus among pregnant women in Anambra state, Nigeria. Shiraz E-Medical Journal 2004; 5(2).
43.	Ezechi OC, Kalejaiye OO, Gab-Okafor CV, et al. Sero-prevalence and factors associated with Hepatitis B and C co-infection in pregnant Nigerian women living with HIV infection. *Pan Afr Med J* 2014; 17: 197.
44.	Fomulu NJ, Morfaw FLI, Torimiro JN, Nana P, Koh MV, William T. Prevalence, correlates and pattern of Hepatitis B among antenatal clinic attenders in Yaounde-Cameroon: Is perinatal transmission of HBV neglected in Cameroon? *BMC Pregnancy and Childbirth* 2013; 13: 1-10.

45. Forbi JC, Campo DS, Purdy MA, et al. Intra-host diversity and evolution of hepatitis C virus endemic to Côte d'Ivoire. *Journal of Medical Virology* 2014; 86(5): 765-71.
46. Fouelifack FY, Metchiem RO, Fouedjio JH, Mbu RE. Seroprevalences and Associated Factors of Viral Infections (HIV, Hepatitis B and C) among Pregnant Women Attending Antenatal Care at the Yaoundé Central Hospital. British *Journal of Medicine & Medical Research* 2017; 20(1): 1-14.
47. Frambo AA, Atashili J, Fon PN, Ndumbe PM. Prevalence of HBsAg and knowledge about hepatitis B in pregnancy in the Buea Health District, Cameroon: a cross-sectional study. *BMC research notes* 2014; 7: 394.
48. Hannachi N, Bahri O, Ben Fredj N, Boukadida J, Triki H. [Risk of vertical transmission of hepatitis B virus in Tunisia]. *Archives de l'Institut Pasteur de Tunis* 2010; 87(1-2): 17-24.
49. Hannachi N, Bahri O, Mhalla S, et al. Hepatitis B virus infection in Tunisian pregnant women: Risk factors and viral DNA levels in HBe antigen negative women. *Pathologie Biologie* 2009; 57(3): e43-e7.
50. Hannachi N, Hidar S, Harrabi I, et al. Seroprevalence and risk factors of hepatitis E among pregnant women in central Tunisia. *Pathologie Biologie* 2011; 59(5): e115-e8.
51. Hashem M, Jhaveri R, Saleh DA, et al. Spontaneous viral load decline and subsequent clearance of chronic hepatitis c virus in postpartum women correlates with favorable interleukin-28B gene allele. *Clinical Infectious Diseases* 2017; 65(6): 999-1005.
52. Hilda AE, Kola OJ, Kolawole OE. Prevalence of anti-hepatitis C virus antibody among pregnant women and blood donors at Bowen University Teaching Hospital, Ogbomoso, Oyo State, Nigeria. *Journal of Immunoassay and Immunochemistry* 2017; 38(2): 221-34.
53. Hoffmann CJ, Mashabela F, Cohn S, et al. Maternal hepatitis B and infant infection among pregnant women living with HIV in South Africa. *Journal of the International AIDS Society* 2014; 17: 18871.
54. Ifeorah IM, Bakarey AS, Adewumi MO, et al. Patterns of serologic markers of hepatitis B virus infection and the risk of transmission among pregnant women in southwestern Nigeria. *Journal of Immunoassay and Immunochemistry* 2017; 38(6): 639-51.
55. Ilboudo D, Simpore J, Ouermi D, et al. Towards the complete eradication of mother-to-child HIV/HBV coinfection at Saint Camille Medical Centre in Burkina Faso, Africa. *Brazilian Journal of Infectious Diseases* 2010; 14(3): 219-24.
56. Jhaveri R, Hashem M, El-Kamary SS, et al. Hepatitis C virus (HCV) vertical transmission in 12-month-old infants born to HCV-infected women and assessment of maternal risk factors. *Open Forum Infectious Diseases* 2015; 2(2) : ofv089.
57. Kassem A, El-Nawawy A, Massoud M, Abou El-Nazar S, Sobhi E. Prevalence of hepatitis C virus (HCV) infection and its vertical transmission in Egyptian pregnant women and their newborns. *Journal of Tropical Pediatrics* 2000; 46(4): 231-3.
58. Kfutwah AKW, Tejiokem MC, Njouom R. A low proportion of HBeAg among HBsAg-positive pregnant women with known HIV status could suggest low perinatal transmission of HBV in Cameroon. *Virology Journal* 2012: 62.
59. Khamis HH, Farghaly AG, Shatat HZ, El-Ghitany EM. Prevalence of hepatitis C virus infection among pregnant women in a rural district in Egypt. *Tropical Doctor* 2016; 46(1): 21-7.
60. Kirbak ALS, Ng'ang'a Z, Omolo J, Idris H, Usman A, Mbabazi WB. Sero-prevalence for hepatitis b virus among pregnant women attending antenatal clinic in Juba teaching hospital, republic of south Sudan. *Pan African Medical Journal* 2017; 26: 72.
61. Kolawole OM, Wahab AA, Adekanle DA, Sibanda T, Okoh AI. Seroprevalence of hepatitis B surface antigenemia and its effects on hematological parameters in pregnant women in Osogbo, Nigeria. *Virology Journal* 2012; 9 : 317.
62. Koneru RK, Mwanga GE, Kisanga F. Seroprevalence of hepatitis B surface antigen and associated factors among pregnant women attending clinic at Mafiga health center, Morogoro, 2014. IMTU Medical Journal 2015; 6(1): 76-82.
63. Lassey AT, Damale NK, Bekoe V, Klufio CA. Hepatitis C virus seroprevalence among mothers delivering at the Korle-Bu Teaching Hospital, Ghana. *East African medical journal* 2004; 81(4): 198-201.
64. Laurenta C, Henzel D, Mulanga-Kabeyaa C, Maertens G, Larouzé B, Delaporte E. Seroepidemiological survey of hepatitis C virus among commercial sex workers and pregnant women in Kinshasa, Democratic Republic of Congo. *International Journal of Epidemiology* 2001; 30(4): 872-7.
65. Luuse A, Dassah S, Lokpo S, et al. Sero-prevalence of hepatitis B surface antigen amongst pregnant women attending an antenatal clinic, Volta region, Ghana. *Journal of Public Health in Africa* 2016; 7(2) : 584.
66. Lennox JA, Mbim E, Uwamere E. Prevalence of Hepatitis B Virus Infection among Pregnant Women Attending Antenatal Clinic at General Hospital Calabar, Cross River State. *International Journal of Sciences* 2015; 4(5): V4201505697.

67. MacLean B, Hess RF, Bonvillain E, et al. Seroprevalence of hepatitis B surface antigen among pregnant women attending the hospital for women & children in Koutiala, Mali. *South African Medical Journal* 2012; 102(1): 47-9.
68. Makuwa M, Caron M, Souquière S, Malonga-Mouelet G, Mahé A, Kazanji M. Prevalence and genetic diversity of hepatitis B and delta viruses in pregnant women in gabon: Molecular evidence that hepatitis delta virus clade 8 originates from and is endemic in Central Africa. *Journal of Clinical Microbiology* 2008; 46(2): 754-6.
69. Mamadou S, Ide M, Ali Maazou AR, Aoula B, Labo S, Bozari M. HIV infection and hepatitis B seroprevalence among antenatal clinic attendees in Niger, West Africa. *HIV/AIDS - Research and Palliative Care* 2012; 4: 1-4.
70. Mansour W, Malick FZF, Sidiya A, et al. Prevalence, risk factors, and molecular epidemiology of hepatitis B and hepatitis delta virus in pregnant women and in patients in Mauritania. *Journal of Medical Virology* 2012; 84(8): 1186-98.
71. Manyahi J, Msigwa Y, Mhimbira F, Majigo M. High sero-prevalence of hepatitis B virus and human immunodeficiency virus infections among pregnant women attending antenatal clinic at Temeke municipal health facilities, Dar es Salaam, Tanzania: A cross sectional study. *BMC Pregnancy and Childbirth* 2017; 17(1): 109.
72. Mavenyengwa RT, Moyo SR, Nordbø SA. Streptococcus agalactiae colonization and correlation with HIV-1 and HBV seroprevalence in pregnant women from Zimbabwe. *European Journal of Obstetrics Gynecology and Reproductive Biology* 2010; 150(1): 34-8.
73. Mbaawuaga EM, Enenebeaku MNO, Okopi JA, Damen JG. Hepatitis B Virus (HBV) infection among pregnant women in Makurdi, Nigeria. *African Journal Biomedical Research* 2008; 11(2): 155-9.
74. Mboto CI, Andy IE, Eni OI, Jewell AP. Prevalence, sociodemographic characteristics and risk factors for hepatitis C infection among pregnant women in Calabar municipality, Nigeria. *Hepatitis Monthly* 2010; 10(2): 116-20.
75. Metaferia Y, Dessie W, Ali I, Amsalu A. Seroprevalence and associated risk factors of hepatitis B virus among pregnant women in southern Ethiopia: a hospital-based cross-sectional study. *Epidemiology and health* 2016; 38: e2016027.
76. Mirambo MM, Mbena PB, Mushi MF, Mtebe M, Seni J, Mshana SE. Prevalence of hepatitis b surface antigen among pregnant women attending antenatal clinic at nyamagana district hospital Mwanza, Tanzania. *Tanzania Journal of Health Research* 2016; 18(1).
77. Molla S, Munshea A, Nibret E. Seroprevalence of hepatitis B surface antigen and anti HCV antibody and its associated risk factors among pregnant women attending maternity ward of Felege Hiwot Referral Hospital, northwest Ethiopia: a cross-sectional study. *Virol J* 2015; 12: 204.
78. Mutagoma M, Balisanga H, Malamba SS, et al. Hepatitis B virus and HIV co-infection among pregnant women in Rwanda. *BMC Infect Dis* 2017; 17(1): 618.
79. Mutagoma M, Balisanga H, Sebuhoro D, et al. Hepatitis C virus and HIV co-infection among pregnant women in Rwanda. *BMC Infect Dis* 2017; 17(1): 167.
80. Ndako JA, Echeonwu GON, Nwankiti, et al. Hepatitis B sero-prevalence among pregnant females in Nigeria. Research Journal of Medical Sciences 2012; 6(3): 129-33.
81. Ndako JA, Echeonwu GON, Olabode AO, et al. Seroprevalence of hepatitis B virus infection amongst pregnant women at the University of Maiduguri Teaching Hospital Maiduguri, Nigeria. *International Journal of Natural and Applied Sciences* 2009; 5(4): 402-6.
82. Ndong-Atome GR, Makuwa M, Njouom R, et al. Hepatitis C virus prevalence and genetic diversity among pregnant women in Gabon, central Africa. *BMC Infect Dis* 2008; 8: 82.
83. Ngaira JAM, Kimotho J, Mirigi I, et al. Prevalence, awareness and risk factors associated with hepatitis b infection among pregnant women attending the antenatal clinic at mbagathi district hospital in Nairobi, Kenya. *Pan African Medical Journal* 2016; 24: 315.
84. Njouom R, Pasquier C, Ayouba A, et al. Hepatitis C virus infection among pregnant women in Yaounde, Cameroon: Prevalence, viremia, and genotypes. *Journal of Medical Virology* 2003; 69(3): 384-90.
85. Njouom R, Pasquier C, Ayouba A, et al. Low risk of mother-to-child transmission of hepatitis C virus in Yaoundé, Cameroon: The ANRS 1262 study. *American Journal of Tropical Medicine and Hygiene* 2005; 73(2): 460-6.
86. Noubiap JJ, Nansseu JR, Ndoula ST, Bigna JJ, Jingi AM, Fokom-Domgue J. Prevalence, infectivity and correlates of hepatitis B virus infection among pregnant women in a rural district of the Far North Region of Cameroon. *BMC public health* 2015; 15: 454.
87. Nwankwo EO, Lawal AM, Abba M. Seroprevalence of Hepatitis C Virus (HCV) antibodies in pregnant women in Anyigba, Kogi State, North Central Nigeria. *African Journal of Clinical and Experimental Microbiology* 2016; 17(3): 219-22.

88. Obi RK, Umeh SC, Okurede OH, Iroagba II. Prevalence of hepatitis B virus infection among pregnant women in an antenatal clinic in Port-Harcourt, Nigeria. *African Journal of Clinical and Experimental Microbiology* 2006; 7(2): 78-82.

89. Obi SN, Onah HE, Ezugwu FO. Risk factors for hepatitis B infection during pregnancy in a Nigerian obstetric population. *Journal of Obstetrics and Gynaecology* 2006; 26(8): 770-2.

90. Ogunlaja OA, Bojuwoye MO, Fawole AA, Adesina AK, Olawumi HO, Ogunlaja IP. Prevalence of hepatitis b virus infection amongst paturients in the University of Ilorin Teaching Hospital. *Nigerian Journal of Gastroenterology and Hepatology* 2015; 7(1): 7-12.

91. Ogunro PS, Adekanle DA, Fadero FF, Ogungbamigbe TO, Oninla SO. Prevalence of anti-hepatitis C virus antibodies in pregnant women and their offspring in a tertiary hospital in Southwestern Nigeria. *Journal of infection in developing countries* 2007; 1(3): 333-6.

92. Okeke TC, Obi SN, Okezie OA, et al. Coinfection with hepatitis B and C viruses among HIV positive pregnant women in Enugu south east, Nigeria. *Nigerian journal of medicine : journal of the National Association of Resident Doctors of Nigeria* 2012; 21(1): 57-60.

93. Okoth F, Mbuthia J, Gatheru Z, et al. Seroprevalence of hepatitis B markers in pregnant women in Kenya. *East African medical journal* 2006; 83(9): 485-93.

94. Okusanya BO, Aigere EOS, Eigbefoh JO, Ikheloa J. Seroprevalence and clinico-epidemiological correlates of hepatitis C viral antibodies at an antenatal booking clinic of a tertiary hospital in Nigeria. *Archives of Gynecology and Obstetrics* 2013; 288(3): 495-500.

95. Oladele OO, Saheed S, Familua F, et al. Seroprevalence of Hepatitis B Surface Antigen and Antibody among Pregnant Women Attending a Tertiary Health Institution in Southwestern Nigeria. *Journal of Dental and Medical Sciences* 2014; 13(3): 67-71.

96. Olaleye OA, Kuti O, Makinde NO, et al. Perinatal transmission of hepatitis B virus infection in Ile-Ife, South Western, Nigeria. *Journal of Neonatal-Perinatal Medicine* 2013; 6(3): 231-6.

97. Olokoba AB, Salawu FK, Danburam A, et al. Hepatitis B virus infection amongst pregnant women in North-Eastern Nigeria - A call for action. *Nigerian Journal of Clinical Practice* 2011; 14(1): 10-3.

98. Oluboyo BO, Ugochukwu VI, Oluboyo AO, et al. Prevalence of Hepatitis B and C viral infections in pregnant women attending antenatal clinic in Nnewi, Nigeria. *European Scientific Journal* 2014; 10(3): 434-41.

99. Onah HE, Obi SN, Agbata TA, Oguanuo TC. Pregnancy outcome in HIV-positive women in Enugu, Nigeria. *Journal of Obstetrics and Gynaecology* 2007; 27(3): 271-4.

100. Onakewhor JU, Okonofua FE. The prevalence of dual human immunodeficiency virus/hepatitis C virus (HIV/HCV) infection in asymptomatic pregnant women in Benin City, Nigeria. *African journal of reproductive health* 2009; 13(2): 97-108.

101. Onakewhor JUE, Offor E, Okonofua FE. Maternal and neonatal seroprevalence of hepatitis B surface antigen (HBsAg) in Benin City, Nigeria. *Journal of Obstetrics and Gynaecology* 2001; 21(6): 583-6.

102. Onwere S, Chigbu B, Aluka C, et al. Risk factors for hepatitis B virus infection during pregnancy in south eastern Nigeria. *East African medical journal* 2012; 89(3): 89-93.

103. Onwuakor CE, Eze VC, Nwankwo IU, Iwu JO. Sero-prevalence of Hepatitis B Surface Antigen (HBsAg) amongst Pregnant Women Attending Antenatal Clinic at the Federal Medical Centre Umuahia, Abia State, Nigeria. *American Journal of Public Health Research* 2014; 2(6): 255-9.

104. Opaleye OO, Igboama MC, Ojo JA, Odewale G. Seroprevalence of HIV, HBV, HCV, and HTLV among Pregnant Women in Southwestern Nigeria. *Journal of Immunoassay and Immunochemistry* 2016; 37(1): 29-42.

105. Osazuwa F, Obinna OV, Chika AF. Sero-epidemiology of human immunodeficency virus, Hepatitis B and C among pregnant women in rural communities of Abaji Area Council, Nigeria. *TAF Preventive Medicine Bulletin* 2012; 11(4): 431-8.

106. Oti BV, Pennap GR, Ngari HR. HBsAg and Anti-HCV Prevalence among Pregnant Women Accessing Antenatal Care in a Tertiary Healthcare Facility in Central Nigeria. *Hepatol Pancreat* Sci 2018, 2:1.

107. Ouermi D, Simpore J, Belem AMG, et al. Co-Infection of Toxoplasma gondii with HBV in HIV-Infected and Uninfected Pregnant Women in Burkina Faso. *Pakistan Journal of Biological Sciences* 2009; 12(17): 1188-93.

108. Owolabi OB, Adesina KT, Fadeyi A, Popoola G. Hepatitis C virus (HCV) seroprevalence, antigenaemia and associated risk factors among pregnant women in Nigeria. *Ethiop Med J* 2015. 53(4): 173-81.

109. Oyinloye SO, Osunkwo M, Taki-Mohd B, Ajayi BB, Lawan MA. Maternal Seroprevalence of Hepatitis B Virus Serologic Markers among Attendees of a Secondary Health Facility in Maiduguri, Nigeria. *British Microbiology Research Journal* 2016; 11(3): 1-7.

110. Pennap GR, Osanga ET, Ubam A. Seroprevalence of hepatitis B surface antigen among pregnant women attending antenatal clinic in federal medical center Keffi, Nigeria. *Research Journal of Medical Sciences* 2011; 5(2): 80-2.

111. Pirillo MF, Bassani L, Germinario EAP, et al. Seroprevalence of hepatitis B and C viruses among HIV-infected pregnant women in Uganda and Rwanda. *Journal of Medical Virology* 2007; 79(12): 1797-801.

112. Pirillo MF, Scarcella P, Andreotti M, et al. Hepatitis B virus mother-to-child transmission among HIV-infected women receiving lamivudine-containing antiretroviral regimens during pregnancy and breastfeeding. *Journal of Viral Hepatitis* 2015; 22(3): 289-96.

113. Rabiu KA, Akinola OI, Adewunmi AA, Omololu OM, Ojo TO. Risk factors for hepatitis B virus infection among pregnant women in Lagos, Nigeria. *Acta Obstetricia et Gynecologica Scandinavica* 2010; 89(8): 1024-8.

114. Ramos JM, Toro C, Reyes F, Amor A, Gutiérrez F. Seroprevalence of HIV-1, HBV, HTLV-1 and Treponema pallidum among pregnant women in a rural hospital in Southern Ethiopia. *Journal of Clinical Virology* 2011; 51(1): 83-5.

115. Randriamahazo TR, Raherinaivo AA, Rakotoarivelo ZH, et al. Prevalence of hepatitis B virus serologic markers in pregnant patients in Antananarivo, Madagascar. *Medecine et Maladies Infectieuses* 2015; 45(1-2): 17-20.

116. Rashid S, Kilewo C, Aboud S. Seroprevalence of hepatitis B virus infection among antenatal clinic attendees at a tertiary hospital in Dar es salaam, Tanzania. *Tanzania Journal of Health Research* 2014; 16(1): 1-8.

117. Rezk M, Omar Z. Deleterious impact of maternal hepatitis-C viral infection on maternal and fetal outcome: a 5-year prospective study. *Archives of Gynecology and Obstetrics* 2017; 296(6): 1097-102.

118. Saleh DA, Shebl F, Abdel-Hamid M, et al. Incidence and risk factors for hepatitis C infection in a cohort of women in rural Egypt. *Transactions of the Royal Society of Tropical Medicine and Hygiene* 2008; 102(9): 921-8.

119. Sangaré L, Sombié R, Combasséré AW, et al. Antenatal transmission of hepatitis B virus in an area of HIV moderate prevalence, Burkina Faso. *Bulletin de la Societe de Pathologie Exotique* 2009; 102(4): 226-9.

120. Sangaré L, Sombié R, Ouedraogo T, et al. Importance of the confirmatory assay for the detection of the HBsAg in the epidemiological studies and in the diagnosis of the viral hepatitis B. *African Journal of Clinical and Experimental Microbiology* 2011. 12(1): 44-8.

121. Sbiti M, Khalki H, Benbella I, Louzi L. Seroprevalence of HBsAg in pregnant women in central Morocco. *Pan African Medical Journal* 2016; 24: 187.

122. Serme AK, Ilboudo PD, Samandoulgou A, Simpore J, Bougouma A, Sombie AR. Prevalence of Hepatitis C virus infection in pregnant women and mother-child transmission in Ouagadougou, Burkina Faso. *Bulletin de la Societe de Pathologie Exotique* 2006; 99(2): 108-9.

123. Shebl FM, El-Kamary SS, Saleh DA, et al. Prospective cohort study of mother-to-infant infection and clearance of hepatitis C in rural Egyptian villages. *Journal of Medical Virology* 2009; 81(6): 1024-31.

124. Sidibe S, Sacko BY, Traoré I. Prevalence of serologic markers of the hepatitis B virus in pregnant women of Bamako, Mali. *Bulletin de la Société de pathologie exotique (1990)* 2001; 94(4): 339-41.

125. Simpore J, Ilboudo D, Karou D, et al. Prevalence of HHV-8 infections associated with HIV, HBV and HCV in pregnant women in Burkina Faso. *Journal of Medical Sciences* 2006; 6(1): 93-8.

126. Simpore J, Ilboudo D, Samandoulougou A, Guardo P, Castronovo P, Musumeci S. HCV and HIV co-infection in pregnant women attending St. Camille Medical Centre in Ouagadougou (Burkina Faso). *Journal of Medical Virology* 2005; 75(2): 209-12.

127. Simpore J, Savadogo A, Ilboudo D, et al. Toxoplasma gondii, HCV, and HBV seroprevalence and co-infection among HIV-positive and -negative pregnant women in Burkina Faso. *Journal of Medical Virology* 2006; 78(6): 730-3.

128. Stoszek SK, Abdel-Hamid M, Narooz S, et al. Prevalence of and risk factors for hepatitis C in rural pregnant Egyptian women. *Transactions of the Royal Society of Tropical Medicine and Hygiene* 2006; 100(2): 102-7.

129. Strand RT, Franque-Ranque M, Bergström S, Weiland O. Infectious aetiology of jaundice among pregnant women in Angola. *Scandinavian Journal of Infectious Diseases* 2003; 35(6-7): 401-3.

130. Tegegne D, Desta K, Tegbaru B, Tilahun T. Seroprevalence and transmission of Hepatitis B virus among delivering women and their new born in selected health facilities, Addis Ababa, Ethiopia: a cross sectional study. *BMC research notes* 2014; 7: 239.

131. Thumbiran NV, Moodley D, Parboosing R, Moodley P. Hepatitis B and HIV co-infection in pregnant women: Indication for routine antenatal hepatitis b virus screening in a high HIV prevalence setting. *South African Medical Journal* 2014; 104(4): 307-9.

132. Ugbebor O, Aigbirior M, Osazuwa F, Enabudoso E, Zabayo O. The prevalence of hepatitis B and C viral infections among pregnant women. *N Am J Med Sci* 2011; 3(5): 238-41.
133. Umare A, Seyoum B, Gobena T, Mariyam TH. Hepatitis B virus infections and associated factors among pregnant women attending antenatal care clinic at deder hospital, eastern Ethiopia. *PLoS ONE* 2016; 11(11).
134. Usanga VU, Abia-Bassey L, Inyang-Etoh PC, Udoh SM, Ani F, Archibong E. Prevalence of sexually transmitted diseases in pregnant and non-pregnant women in Calabar, cross river state, Nigeria. *Internet Journal of Gynecology and Obstetrics* 2011; 14(2).
135. Utoo BT, Utoo PM, Bassey SN, Ojinnaka VC. Hepatitis C Virus and Human Immunodeficiency Virus Co-Infection among Pregnant Women in South-South, Nigeria. *Jos Journal of Medicine* 2011; 5(2): 31-35.
136. Utoo BT. Hepatitis B surface antigenemia (HBsAg) among pregnant women in Southern Nigeria. *African Health Sciences* 2013; 13(4): 1139-43.
137. Völker F, Cooper P, Bader O, et al. Prevalence of pregnancy-relevant infections in a rural setting of Ghana. *BMC Pregnancy and Childbirth* 2017; 17: 172.
138. Wurie IM, Wurie AT, Gevao SM. Sero-prevalence of hepatitis B virus among middle to high socio-economic antenatal population in Sierra Leone. *West African Journal of Medicine* 2005; 24(1): 18-20.
139. Ya'Aba Y, Isu NR, Mohammed SB, et al. Prevalence of Hepatitis C Virus (HCV) and Human Immunodeficiency Virus (HIV) Co-infection among pregnant women attending antenatal clinics in Abuja, Nigeria. *Journal of Phytomedicine and Therapeutics* 2009; 14: 45-8.
140. Yakasai IA, Ayyuba R, Abubakar IS, Ibrahim SA. Sero-prevalence of Hepatitis B Virus Infection and its Risk factors among Pregnant Women Attending Antenatal Clinic at Aminu Kano Teaching Hospital, Kano, Nigeria. *Journal of Basic and Clinical Reproductive Sciences* 2012; 1(1-2): 49-55.
141. Yohanes T, Zerdo Z, Chufamo N. Seroprevalence and Predictors of Hepatitis B Virus Infection among Pregnant Women Attending Routine Antenatal Care in Arba Minch Hospital, South Ethiopia. *Hepatitis Research and Treatment* 2016; 2016: 9290163.
142. Zahran KM, Badary MS, Agban MN, Abdel Aziz NHR. Pattern of hepatitis virus infection among pregnant women and their newborns at the Women's Health Center of Assiut University, Upper Egypt. *International Journal of Gynecology and Obstetrics* 2010; 111(2): 171-4.
143. Zeba MTA, Karou SD, Sagna T, et al. HCV prevalence and co-infection with HIV among pregnant women in Saint Camille Medical Centre, Ouagadougou. *Tropical Medicine and International Health* 2011; 16(11): 1392-6.
144. Zenebe Y, Mulu W, Yimer M, Abera B. Sero-prevalence and risk factors of hepatitis C virus infection among pregnant women in Bahir Dar city, Northwest Ethiopia: Cross sectional study. *Pan African Medical Journal* 2015; 21 : 158.
145. Zenebe Y, Mulu W, Yimer M, Abera B. Sero-prevalence and risk factors of hepatitis B virus and human immunodeficiency virus infection among pregnant women in Bahir Dar city, Northwest Ethiopia: a cross sectional study. *BMC Infect Dis* 2014; 14: 118.

Supplemental Table 3 : ***List of countries***

**Countries**	**N Studies**
Algeria	1
Angola	1
Benin	1
Burkina Faso	10
Cameroon	10
Democratic Republic of the Congo	1
Egypt	13
Ethiopia	9
Gabon	2
Ghana	6
Ivory Coast	1
Kenya	2
Libya	1
Madagascar	1
Malawi	4
Mali	2
Mauritania	1
Morocco	1
Niger	1
Nigeria	53
Republic of South Sudan	1
Rwanda	1
Sierra Leone	1
South Africa	7
Sudan	1
Tanzania	4
Togo	1
Tunisia	3
Uganda	1
Zimbabwe	1
Uganda, Rwanda	1

*Supplemental Table 4 : **Individual characteristics of included studies***

Study	Year of publication	Country	Design	N Centers	Area	Setting	Timing of data collection	Period of data collection	Sampling	Mean or median age, years	Age limit, years	%HBV Vaccination	%HIV-infected	%Secondary level of education or higher	%Single	%Married	%Divorced	%Widow	%History of transfusion	%Residing in Rural	%History of multiple sexual partners	%Histroty of Surgery	%Primigravida	%Scarification	%Tatoos	%Piercing	%History of circumcision	%History of abortion	%Dental procedure	%Traditional birth attendance	%Polygamous	Sample	Risk of bias
AbdulKawi, 2010	2010	Egypt	Cross-sectional	Single-center	Urban, Rural	Antenatal care	Prospective	2003-2008	Systematic	25.3	16-45	NR	NR	NR	NR	NR	NR	NR	4.66	80.96	NR	28.02	73	NR	NR	NR	NR	NR	NR	NR	NR	1224	Low
Adabara, 2012	2012	Nigeria	Cross-sectional	Single-center	Urban	Antenatal care	Prospective	2010	Consecutive	NR	NR	NR	NR	NR	NR	NR	NR	NR	NR	NR	NR	NR	NR	NR	NR	NR	NR	NR	NR	NR	NR	200	Low
Adegbesan,2015	2015	Nigeria	Cross-sectional	Single-center	Urban	Antenatal care	Prospective	NR	Consecutive	28.5	18-44	NR	NR	28.5	8	92	NR	NR	2.2	NR	27.3	19.3	34.7	NR	NR	NR	NR	NR	NR	NR	NR	150	Low
Adesina, 2016	2016	Nigeria	Cross-sectional	Single-center	Urban, Rural	PMTCT	Prospective	2006-2013	Consecutive	29.2	NR	NR	100	63.7	7.9	92.1	NR	NR	1.1	NR	NR	NR	NR	NR	NR	NR	NR	NR	NR	NR	NR	1433	Low
Aidaoui, 2008	2008	Algeria	Cross-sectional	Multi-center	Urban	Antenatal care	Prospective	2003-2004	Systematic	31.00	NR	0.53	0.53	0.53	1	99	0	0	0.53	NR	NR	0.53	2.7	NR	NR	NR	NR	25	NR	NR	NR	3032	Low
Ajugwo, 2017	2017	Nigeria	Cross-sectional	Single-center	Urban, Rural	Other hospital based (not antenatal care)	Prospective	NR	Consecutive	NR	NR	NR	NR	NR	NR	NR	NR	NR	NR	NR	NR	NR	NR	NR	NR	NR	NR	NR	NR	NR	NR	200	Moderate
Alegbeleye, 2013	2013	Nigeria	Cross-sectional	Single-center	Urban, Rural	Antenatal care	Prospective	2011	Consecutive	33.5	20-45	0	0	97.6	0	100	0	0	8.4	NR	NR	21.2	42.8	NR	22.4	NR	NR	46.8	24.8	NR	2.8	250	Low
Aluor, 2016	2016	Nigeria	Cross-sectional	Multi-center	Urban, Rural	Antenatal care, Community	Prospective	2012-2013	Random	NR	11-45	NR	NR	13.55	NR	NR	NR	NR	4.84	NR	NR	NR	NR	NR	NR	NR	NR	NR	NR	NR	NR	301	Low
Anaedobe, 2015	2015	Nigeria	Cross-sectional	Single-center	Urban, Rural	Antenatal care	Prospective	2013	Consecutive	32.00	22-44	0	20.56	97.2	4.4	95.6	NR	NR	13.9	NR	39.4	30	29.4	NR	NR	NR	NR	NR	NR	NR	4.4	180	Low
Andersson, 2012	2012	South Africa	Cross-sectional	Single-center	Urban	Antenatal care	Retrospective	2008-2009	Systematic	28.00	NR	NR	100	NR	NR	NR	NR	NR	NR	NR	NR	NR	NR	NR	NR	NR	NR	NR	NR	NR	NR	3089	Moderate
Andreotti, 2014	2014	Malawi	Baseline data of a Cohort study	Single-center	Urban, Rural	Other hospital based (not antenatal care)	Prospective	2008-2009	Consecutive	27.00	16-	NR	100	NR	NR	NR	NR	NR	NR	NR	NR	NR	NR	NR	NR	NR	NR	NR	NR	NR	NR	309	Low
Apea-Kubi, 2006	2006	Ghana	Cross-sectional	Single-center	Urban	Antenatal care	Prospective	2000-2001	Consecutive	29.6	NR	NR	4.1	62.9	8.8	86.4	NR	NR	NR	NR	NR	NR	40.2	NR	NR	NR	NR	NR	NR	NR	NR	294	Low
Araya, 2017	2017	Ethiopia	Cross-sectional	Multi-center	Urban, Rural	Antenatal care	Prospective	2015	Consecutive	25.5	18-43	NR	4.6	62.2	1.8	95.7	2.1	0.3	NR	28.3	11.3	5.5	NR	38.1	5.5	5.5	5.5	5.5	8.5	NR	NR	328	Low
Bayo, 2014	2014	Uganda	Cross-sectional	Multi-center	Rural	Antenatal care	Prospective	2012-2013	Systematic	24.00	13-43	NR	9.3	44.4	3.8	96.2	NR	NR	NR	NR	NR	NR	23.4	89.2	NR	NR	NR	NR	NR	NR	28.2	397	Low
Burnett, 2007	2007	South Africa	Case-control (group of pregnant women)	Multi-center	Urban, Rural	Antenatal care	Retrospective	1999-2001	Consecutive	NR	NR	NR	50	NR	NR	NR	NR	NR	NR	NR	NR	NR	NR	NR	NR	NR	NR	NR	NR	NR	NR	1420	Moderate

Study	Year of publication	Country	Design	N Centers	Area	Setting	Timing of data collection	Period of data collection	Sampling	Mean or median age, years	Age limit, years	%HBV Vaccination	%HIV-infected	%Secondary level of education or higher	%Single	%Married	%Divorced	%Widow	%History of transfusion	%Residing in Rural	%History of multiple sexual partners	%Histroty of Surgery	%Primigravida	%Scarification	%Tatoos	%Piercing	%History of circumcision	%History of abortion	%Dental procedure	%Traditional birth attendance	%Polygamous	Sample	Risk of bias
Candotti, 2007	2007	Ghana	Cross-sectional	Multi-center	Urban, Rural	Antenatal care, Other hospital based (not antenatal care)	Prospective	NR	Consecutive	27.00	15-48	NR	NR	NR	NR	NR	NR	NR	NR	NR	NR	NR	NR	NR	NR	NR	NR	NR	NR	NR	NR	1368	Moderate
Chasela, 2012	2012	Malawi	Cross-sectional	Multi-center	Urban, Rural	Antenatal care	Prospective	NR	Random	25.00	14-45	NR	100	NR	NR	NR	NR	NR	NR	NR	NR	NR	NR	NR	NR	NR	NR	NR	NR	NR	NR	2041	Moderate
Chasela, 2014	2014	Malawi	Cross-sectional	Multi-center	Urban, Rural	Antenatal care	Prospective	2004-2009	Random	25.00	14-	NR	100	NR	NR	NR	NR	NR	NR	NR	NR	NR	NR	NR	NR	NR	NR	NR	NR	NR	NR	2048	Low
Cho, 2012	2012	Ghana	Cross-sectional	Multi-center	Urban, Rural	Other hospital based (not antenatal care)	Prospective	2008-2009	Consecutive	NR	NR	NR	6.0	14.1	NR	NR	NR	NR	NR	NR	NR	NR	NR	NR	NR	NR	NR	NR	NR	NR	NR	1250	Low
Chotun, 2017	2017	South Africa	Baseline data of a Cohort study	Single-center	Urban, Rural	PMTCT	Prospective	2014	Consecutive	26.1	NR	NR	0	NR	NR	NR	NR	NR	NR	NR	NR	NR	NR	NR	NR	NR	NR	NR	NR	NR	NR	134	Low
Collenberg, 2006	2009	Burkina Faso	Cross-sectional	Multi-center	Urban, Rural	Antenatal care, PMTCT	Prospective	2003-2004	Consecutive	25.5	16-46	NR	3.98	NR	23.7	56.2	NR	NR	NR	NR	NR	NR	34	NR	NR	NR	NR	NR	NR	NR	NR	492	Low
Desalegn, 2016	2016	Ethiopia	Cross-sectional	Single-center	Urban, Rural	Antenatal care	Prospective	2014	Consecutive	26.00	15-39	NR	4.2	42.4	NR	98.1	NR	NR	3.3	4.7	NR	18.1	NR	NR	2.5	NR	NR	2.1	NR	NR	NR	215	Low
Dionne-Odom, 2016	2016	Cameroon	Cross-sectional	Multi-center	Urban, Rural	Antenatal care	Retrospective	2014	Systematic	26.00	14-55	NR	6	NR	NR	NR	NR	NR	NR	NR	NR	NR	NR	NR	NR	NR	NR	NR	NR	NR	NR	7069	Low
Ducancelle, 2013	2013	Cameroon	Baseline data of a Cohort study	Single-center	Rural	Antenatal care	Retrospective	2009-2010	Consecutive	NR	NR	0.0	NR	NR	NR	NR	NR	NR	NR	100	NR	NR	NR	NR	NR	NR	NR	NR	NR	NR	NR	1267	Low
Eke, 2011	2011	Nigeria	Cross-sectional	Multi-center	Urban, Rural	Antenatal care	Prospective	2009	Random	24.3	14-45	0.0	11.9	64.2	4.2	95.8	NR	NR	16.67	NR	29.17	23.3	NR	4.17	NR	NR	NR	NR	NR	NR	NR	480	Low
El-Kamary, 2015	2015	Egypt	Cross-sectional	Single-center	Urban	Antenatal care	Prospective	2012-2013	Consecutive	27.4	16-45	NR	NR	NR	NR	NR	NR	NR	1.3	NR	NR	62.9	22.1	NR	NR	NR	NR	NR	NR	NR	NR	1250	Low
El-Karasky, 2014	2014	Egypt	Cross-sectional	Single-center	Urban, Rural	Other hospital based (not antenatal care)	Prospective	2010-2011	Consecutive	27.00	NR	3.4	NR	NR	NR	NR	NR	NR	8.55	NR	NR	58.97	NR	NR	NR	NR	96.58	NR	55.55	NR	NR	2000	Moderate
El-Magrahe, 2010	2010	Libya	Cross-sectional	Single-center	Urban, Rural	Other hospital based (not antenatal care)	Prospective	2001-2002	Consecutive	28.00	21-35	0	NR	NR	NR	NR	NR	NR	NR	NR	NR	NR	NR	NR	NR	NR	NR	NR	NR	NR	NR	1500	Low
Elrazek, 2017	2017	Egypt	Baseline data of a Cohort study	Single-center	Urban, Rural	Antenatal care	Retrospective	2015	Random	26.4	18-43	NR	NR	NR	NR	NR	NR	NR	NR	NR	NR	NR	18.63	NR	NR	NR	NR	NR	NR	NR	NR	3000	Low

Study	Year of publication	Country	Design	N Centers	Area	Setting	Timing of data collection	Period of data collection	Sampling	Mean or median age, years	Age limit, years	%HBV Vaccination	%HIV-infected	%Secondary level of education or higher	%Single	%Married	%Divorced	%Widow	%History of transfusion	%Residing in Rural	%History of multiple sexual partners	%Histroty of Surgery	%Primigravida	%Scarification	%Tatoos	%Piercing	%History of circumcision	%History of abortion	%Dental procedure	%Traditional birth attendance	%Polygamous	Sample	Risk of bias
Elshreikh, 2007	2007	Sudan	Case-control (group of pregnant women)	Single-center	Urban, Rural	Antenatal care	Prospective	2006	Consecutive	27.3	21-34	NR	NR	60	NR	NR	NR	NR	8.8	NR	NR	NR	17.5	9	3	NR	NR	NR	NR	NR	NR	728	Low
Enow-Tanjong, 2016	2016	Cameroon	Cross-sectional	Multi-center	Urban, Rural	Antenatal care	Prospective	2010	Consecutive	26.0	15-47	NR	8.4	69.9	35.5	63.1	0.0	1.5	NR	NR	NR	NR	34.7	NR	NR	NR	NR	NR	NR	NR	NR	406	Low
Ephraim, 2015	2015	Ghana	Cross-sectional	Single-center	Rural	Antenatal care	Prospective	2012-2013	Consecutive	NR	10-40	NR	NR	24	NR	NR	NR	NR	NR	NR	NR	NR	NR	NR	NR	NR	NR	NR	NR	NR	NR	168	Low
Erhabor, 2017	2017	Nigeria	Cross-sectional	Single-center	Rural	Antenatal care	Prospective	NR	Consecutive	NR	18-40	NR	NR	0.85	NR	NR	NR	NR	5.13	100	NR	NR	NR	NR	NR	NR	NR	NR	NR	NR	NR	117	Low
Esan, 2014	2014	Nigeria	Cross-sectional	Single-center	Urban, Rural	Antenatal care	Prospective	2012-2013	Consecutive	NR	15-41	NR	NR	NR	NR	NR	NR	NR	NR	NR	NR	NR	NR	NR	NR	NR	NR	NR	NR	NR	NR	649	Low
Evelyn,2009	2009	Nigeria	Cross-sectional	Single-center	Urban	Antenatal care	Prospective	NR	Consecutive	NR	NR	NR	NR	NR	NR	NR	NR	NR	NR	NR	NR	NR	NR	NR	NR	NR	NR	NR	NR	NR	NR	465	Moderate
Ezechi, 2014	2014	Nigeria	Cross-sectional	Single-center	Urban	PMTCT	Prospective	2006-2011	Consecutive	29.5	14-44	NR	100	81.7	NR	81.5	1.4	2.4	4.47	NR	NR	NR	65.37	NR	NR	NR	NR	63	NR	NR	NR	2391	Low
Ezegbudo, 2004	2004	Nigeria	Cross-sectional	Multi-center	Urban, Rural	Antenatal care	Prospective	2002-2003	Random	NR	16-50	NR	8.6	NR	NR	NR	NR	NR	NR	NR	NR	NR	NR	NR	NR	NR	NR	NR	NR	NR	NR	1120	Low
Fomulu, 2013	2013	Cameroon	Cross-sectional	Multi-center	Urban	Antenatal care	Prospective	2011-2012	Consecutive	27.6	15-43	2.7	8.4	33.1	18.9	61.7	NR	NR	4.8	NR	NR	13	31.8	18.54	NR	NR	NR	38	NR	NR	NR	959	Low
Forbi, 2014	2014	Ivory Coast	Baseline data of a Cohort study	Multi-center	Urban, Rural	Community	Retrospective	1995	Consecutive	NR	NR	NR	NR	NR	NR	NR	NR	NR	NR	NR	NR	NR	NR	NR	NR	NR	NR	NR	NR	NR	NR	608	Moderate
Fouelifack, 2017	2017	Cameroon	Cross-sectional	Single-center	Urban	Antenatal care	Prospective	2016	Consecutive	27.99	15-47	4.2	13.1	91.66	31.67	68.33	0	0	6.94	2.5	10	13.05	58.61	29.72	8.61	2.5	NR	NR	26.11	NR	NR	360	Low
Frambo, 2014	2014	Cameroon	Cross-sectional	Multi-center	Urban, Rural	Antenatal care	Prospective	2012	Consecutive	25.7	17-42	NR	NR	80.1	35.1	65	NR	NR	6.9	NR	NR	12	31.8	60.5	2.7	90.8	NR	32.2	NR	NR	2.9	176	Low
Hannachi, 2009	2009	Tunisia	Cross-sectional	Multi-center	Urban, Rural	Antenatal care	Prospective	2007	Consecutive	29.00	18-43	1.4	NR	NR	NR	NR	NR	NR	2.13	NR	NR	9	NR	6.89	NR	NR	NR	NR	NR	NR	NR	2303	Moderate
Hannachi, 2010	2010	Tunisia	Cross-sectional	Multi-center	Urban, Rural	Antenatal care	Prospective	2007-2008	Consecutive	29.00	14-45	3	NR	NR	NR	NR	NR	NR	NR	NR	NR	NR	NR	NR	NR	NR	NR	NR	NR	NR	NR	2709	Moderate
Hannachi, 2011	2011	Tunisia	Cross-sectional	Single-center	Urban	Antenatal care	Prospective	2006	Consecutive	30.08	17-52	NR	NR	NR	NR	NR	NR	NR	5.69	33.2	NR	31.93	61.88	NR	26.73	NR	NR	61.88	NR	NR	NR	404	Low
Hashem, 2017	2017	Egypt	Baseline data of a Cohort study	Single-center	Urban	Antenatal care	Prospective	2012-2014	Consecutive	31.7	NR	NR	NR	NR	NR	NR	NR	NR	7.7	NR	NR	65.4	7.7	NR	0.0	NR	NR	32.7	80.8	NR	NR	2514	Moderate
Hilda, 2016	2016	Nigeria	Cross-sectional	Single-center	Urban, Rural	Antenatal care	Prospective	NR	Consecutive	31.4	16-55	NR	NR	82.61	0.0	100	NR	NR	2.9	NR	NR	15.9	57.2	NR	NR	NR	NR	NR	NR	NR	4.35	138	Low

Study	Year of publication	Country	Design	N Centers	Area	Setting	Timing of data collection	Period of data collection	Sampling	Mean or median age, years	Age limit, years	%HBV Vaccination	%HIV-infected	%Secondary level of education or higher	%Single	%Married	%Divorced	%Widow	%History of transfusion	%Residing in Rural	%History of multiple sexual partners	%Histroty of Surgery	%Primigravida	%Scarification	%Tatoos	%Piercing	%History of circumcision	%History of abortion	%Dental procedure	%Traditional birth attendance	%Polygamous	Sample	Risk of bias
Hoffmann, 2014	2014	South Africa	Case-control (group of pregnant women)	Multi-center	Urban	Antenatal care	Prospective	NR	Consecutive	29.00	18-	NR	100	NR	NR	NR	NR	NR	NR	NR	NR	NR	NR	NR	NR	NR	NR	NR	NR	NR	NR	1891	Low
Ifeorah, 2017	2017	Nigeria	Cross-sectional	Multi-center	Urban, Rural	Antenatal care	Prospective	2012-2013	Consecutive	NR	NR	8.9	NR	NR	NR	NR	NR	NR	NR	NR	NR	NR	NR	NR	NR	NR	NR	NR	NR	NR	NR	272	Low
Ilboudo, 2010	2010	Burkina Faso	Cross-sectional	Single-center	Urban, Rural	Antenatal care, Other hospital based (not antenatal care)	Prospective	2007-2009	Consecutive	28.1	18-41	NR	100	NR	NR	NR	NR	NR	NR	NR	NR	NR	NR	NR	NR	NR	NR	NR	NR	NR	NR	115	Low
Jhaveri, 2015	2015	Egypt	Case-control (group of pregnant women)	Single-center	Urban	Antenatal care	Prospective	2012-2014	Consecutive	32.00	26-37	NR	NR	NR	NR	NR	NR	NR	NR	NR	NR	NR	NR	NR	NR	NR	NR	NR	NR	NR	NR	2514	Moderate
Kassem, 2000	2000	Egypt	Cross-sectional	Single-center	Urban, Rural	Other hospital based (not antenatal care)	Prospective	1996	Random	27.6	25-41	NR	0.0	NR	NR	NR	NR	NR	9	NR	NR	12	NR	NR	NR	NR	NR	13	NR	NR	NR	100	Low
Kfutwah, 2012	2012	Cameroon	Baseline data of a Cohort study	Single-center	Urban, Rural	Antenatal care	Retrospective	2000-2003	Consecutive	26.17	NR	NR	46.3	NR	NR	NR	NR	NR	NR	NR	NR	NR	NR	NR	NR	NR	NR	NR	NR	NR	NR	650	Low
Khamis, 2016	2016	Egypt	Cross-sectional	Multi-center	Urban, Rural	Antenatal care	Prospective	NR	Consecutive	25.87	16-45	NR	NR	33.3	NR	NR	NR	NR	4.16	NR	NR	NR	NR	NR	NR	NR	NR	NR	NR	NR	NR	360	Low
Kirbak, 2017	2017	Republic of South Sudan	Cross-sectional	Single-center	Urban, Rural	Antenatal care	Prospective	2012-2013	Consecutive	NR	15-44	1.8	NR	6.4	1.8	73	12.9	NR	6.18	35.36	18.6	33.33	24.29	21.8	NR	NR	NR	17.5	NR	NR	32.4	280	Low
Kolawole, 2012	2012	Nigeria	Cross-sectional	Single-center	Rural	Antenatal care	Prospective	2010-2011	Consecutive	NR	NR	4	NR	49.5	NR	NR	NR	NR	2	NR	NR	NR	44.5	NR	35.5	NR	NR	18.5	NR	NR	9	200	Low
Koneru, 2015	2015	Tanzania	Cross-sectional	Single-center	Urban, Rural	Antenatal care	Prospective	2014	Random	24.7	14-40	NR	4.0	25.8	11.9	87.7	0.4	0.0	2.4	NR	37.3	9.1	3.3	NR	1.2	86.9	NR	12.3	28.1	NR	NR	252	Low
Lassey, 2004	2004	Ghana	Cross-sectional	Single-center	Urban, Rural	Other hospital based (not antenatal care)	Prospective	2001	Random	NR	NR	NR	2.7	NR	NR	NR	NR	NR	NR	NR	NR	NR	51.4	NR	NR	NR	NR	NR	NR	NR	NR	638	Low
Laurebt, 2001	2001	Democratic Republic of Congo	Cross-sectional	Multi-center	Urban, Rural	Antenatal care, Other hospital based	Retrospective	1990	Random	25.6	14-45	NR	2.8	NR	22.6	77.4	NR	NR	NR	NR	20	NR	NR	NR	NR	NR	NR	NR	NR	NR	20.8	1092	Low

Study	Year of publication	Country	Design	N Centers	Area	Setting	Timing of data collection	Period of data collection	Sampling	Mean or median age, years	Age limit, years	%HBV Vaccination	%HIV-infected	%Secondary level of education or higher	%Single	%Married	%Divorced	%Widow	%History of transfusion	%Residing in Rural	%History of multiple sexual partners	%Histroty of Surgery	%Primigravida	%Scarification	%Tatoos	%Piercing	%History of circumcision	%History of abortion	%Dental procedure	%Traditional birth attendance	%Polygamous	Sample	Risk of bias
						(not antenatal care)																										
Lennox, 2015	2015	Nigeria	Cross-sectional	Single-center	Urban, Rural	Antenatal care	Prospective	2012	Random	NR	15-42	NR	NR	NR	NR	NR	NR	NR	NR	NR	NR	NR	NR	NR	NR	NR	NR	NR	NR	NR	NR	150	Low
Luuse, 2016	2016	Ghana	Cross-sectional	Single-center	Urban, Rural	Antenatal care	Prospective	2016	Consecutive	27.7	15-50	NR	NR	NR	27.4	72.6	0	0	NR	NR	NR	NR	25.96	NR	NR	NR	NR	NR	NR	NR	NR	208	Low
MacLean, 2012	2012	Mali	Cross-sectional	Single-center	Urban, Rural	Antenatal care, Other hospital based (not antenatal care)	Prospective	2008-2009	Random	NR	13-46	NR	4.78	NR	NR	NR	NR	NR	NR	NR	NR	NR	NR	NR	NR	NR	96.4	NR	NR	NR	NR	3659	Low
Makuwa, 2007	2007	Gabon	Baseline data of a Cohort study	Multi-center	Urban, Rural	Antenatal care	Prospective	2005	Consecutive	25.00	14-40	NR	NR	NR	NR	NR	NR	NR	NR	NR	NR	NR	NR	NR	NR	NR	NR	NR	NR	NR	NR	1186	Low
Malungu Ngaira, 2016	2016	Kenya	Cross-sectional	Single-center	Urban	Antenatal care	Prospective	2014	Random	26.7	16-49	3.5	NR	70.65	12.2	87.8	NR	NR	4.53	NR	5.6	12.54	46.6	NR	NR	NR	NR	16.72	NR	NR	5.92	287	Low
Mamadou, 2012	2012	Niger	Cross-sectional	Multi-center	Urban, Rural	Antenatal care	Prospective	2008	Consecutive	NR	15-49	NR	2.0	9.7	NR	NR	NR	NR	NR	NR	NR	NR	NR	NR	NR	NR	NR	NR	NR	NR	NR	495	Low
Mansour, 2012	2012	Mauritania	Cross-sectional	Single-center	Urban, Rural	Other hospital based (not antenatal care)	Prospective	2008-2009	Consecutive	26.5	14-47	NR	NR	12.5	0.1	99.1	NR	NR	2.5	45.8	NR	NR	NR	NR	NR	NR	NR	NR	NR	NR	4.4	1020	Low
Manyahi, 2017	2017	Tanzania	Cross-sectional	Multi-center	Urban	Antenatal care	Prospective	2014	Consecutive	25.00	18-40	NR	17.3	46.2	20.9	78.7	0.4	0	NR	NR	NR	NR	NR	NR	NR	NR	NR	NR	NR	NR	NR	249	Low
Mavenyengwa, 2010	2010	Zimbabwe	Cross-sectional	Multi-center	Urban, Rural	Antenatal care	Prospective	2003-2005	Consecutive	NR	16-45	NR	42	NR	NR	NR	NR	NR	NR	NR	NR	NR	NR	NR	NR	NR	NR	NR	NR	NR	NR	418	Low
Mbaawuaga, 2008	2008	Nigeria	Cross-sectional	Multi-center	Urban, Rural	Antenatal care	Prospective	2005	Consecutive	NR	10-49	NR	NR	NR	NR	NR	NR	NR	NR	NR	NR	NR	NR	NR	NR	NR	NR	NR	NR	NR	NR	300	Low
Mboto, 2010	2010	Nigeria	Cross-sectional	Multi-center	Urban, Rural	Antenatal care	Prospective	2005	Consecutive	29.3	15-39	NR	NR	NR	5.3	93.3	1.4	NR	5.3	NR	NR	2.6	NR	NR	0.2	NR	15.2	NR	NR	NR	9.9	506	Low
Metaferia, 2016	2016	Ethiopia	Cross-sectional	Single-center	Urban, Rural	Antenatal care	Prospective	2015	Consecutive	26.0	18-39	0	5.2	72.8	NR	NR	NR	NR	3.3	16	14.5	NR	NR	NR	18.2	NR	75.1	27.5	NR	17.5	NR	269	Low
Mirambo,2016	2016	Tanzania	Cross-sectional	Single-center	Urban, Rural	Antenatal care	Prospective	2014	Consecutive	23.00	NR	NR	NR	NR	NR	95.7	4.3	NR	NR	NR	NR	NR	61.6	NR	NR	NR	NR	NR	NR	NR	NR	211	Low
Molla, 2015	2015	Ethiopia	Cross-sectional	Single-center	Urban, Rural	Antenatal care	Prospective	2013-2014	Consecutive	26.96	25-29	NR	NR	31.8	5.5	93.2	1.3	NR	NR	11.5	NR	NR	NR	NR	NR	NR	NR	NR	NR	NR	NR	384	Low
Mu,2009	2009	Nigeria	Cross-sectional	Single-center	Urban	Antenatal care	Prospective	2003	Consecutive	NR	NR	NR	3	NR	NR	NR	NR	NR	7	NR	NR	NR	NR	NR	NR	NR	NR	NR	NR	NR	NR	200	Low
Mutagoma, 2017	2017	Rwanda	Baseline data of a Cohort study	Multi-center	Urban, Rural	Antenatal care, PMTCT	Prospective	2011	Consecutive	NR	15-49	NR	3.32	11.38	11.14	86.54	1.60	NR	NR	49.72	NR	NR	30.12	NR	NR	NR	NR	NR	NR	NR	NR	12903	Low

Study	Year of publication	Country	Design	N Centers	Area	Setting	Timing of data collection	Period of data collection	Sampling	Mean or median age, years	Age limit, years	%HBV Vaccination	%HIV-infected	%Secondary level of education or higher	%Single	%Married	%Divorced	%Widow	%History of transfusion	%Residing in Rural	%History of multiple sexual partners	%Histroty of Surgery	%Primigravida	%Scarification	%Tatoos	%Piercing	%History of circumcision	%History of abortion	%Dental procedure	%Traditional birth attendance	%Polygamous	Sample	Risk of bias
Ndako, 2009	2009	Nigeria	Cross-sectional	Single-center	Urban, Rural	Antenatal care	Prospective	NR	Consecutive	NR	20-49	NR	NR	74	NR	NR	NR	NR	0.15	NR	NR	27.5	NR	NR	64.5	NR	NR	NR	NR	NR	NR	200	Low
Ndako, 2012	2012	Nigeria	Cross-sectional	Single-center	Urban, Rural	Antenatal care	Prospective	NR	Consecutive	NR	13-49	NR	NR	NR	NR	NR	NR	NR	8.33	NR	11.11	38.89	NR	NR	NR	NR	1.67	NR	NR	NR	NR	180	Moderate
Ngong-atome, 2008	2008	Gabon	Cross-sectional	Multi-center	Urban	Antenatal care	Prospective	2005	Consecutive	NR	14-	NR	NR	NR	NR	NR	NR	NR	NR	NR	NR	NR	NR	NR	NR	NR	NR	NR	NR	NR	NR	947	Low
Njouom, 2003	2003	Cameroon	Cross-sectional	Multi-center	Urban	Antenatal care	Prospective	200-2001	Consecutive	26.00	14-43	NR	NR	NR	NR	NR	NR	NR	NR	NR	NR	NR	NR	NR	NR	NR	NR	NR	NR	NR	NR	1494	Low
Njouom, 2005	2005	Cameroon	Case-control (group of pregnant women)	Multi-center	Urban	Antenatal care	Prospective	2001-2003	Systematic	25.6	13-46	NR	8	NR	NR	NR	NR	NR	NR	NR	NR	NR	NR	NR	NR	NR	NR	NR	NR	NR	NR	5008	Low
Noubiap, 2015	2015	Cameroon	Cross-sectional	Multi-center	Rural	Antenatal care	Prospective	2013-2014	Systematic	24.4	15-40	1.2	2.5	20	2.5	97.2	0	0.3	1.2	100	NR	2.2	41.5	19.2	NR	NR	0.9	12.9	3.1	NR	NR	325	Low
Nwankwo, 2016	2016	Nigeria	Cross-sectional	Multi-center	Urban, Rural	Other hospital based (not antenatal care)	Prospective	2013	Consecutive	NR	11-50	NR	NR	NR	NR	NR	NR	NR	6.15	NR	0.0	NR	NR	NR	4.62	NR	NR	NR	NR	NR	NR	130	Low
Obi, 2006	2006	Nigeria	Cross-sectional	Multi-center	Urban, Rural	Antenatal care	Prospective	2005	Consecutive	NR	NR	NR	NR	NR	NR	NR	NR	NR	NR	NR	NR	NR	NR	NR	NR	NR	NR	NR	NR	NR	NR	1499	Low
Obi, 2006	2006	Nigeria	Cross-sectional	Single-center	Urban, Rural	Antenatal care, Other hospital based (not antenatal care)	Prospective	2000-2004	Consecutive	NR	15-45	NR	NR	NR	NR	NR	NR	NR	NR	NR	NR	NR	NR	NR	NR	NR	NR	NR	NR	NR	NR	10032	Low
Ogundeyi, 2017	2017	Nigeria	Cross-sectional	Single-center	Urban, Rural	Antenatal care	Prospective	2015	Consecutive	NR	NR	NR	NR	54	3	97	0	0	NR	NR	NR	NR	NR	13	4	NR	NR	NR	NR	NR	NR	100	Low
Ogunlaja, 2015	2015	Nigeria	Cross-sectional	Single-center	Urban, Rural	Antenatal care	Prospective	2012	Consecutive	31.2	NR	NR	8.9	NR	NR	NR	NR	NR	NR	NR	NR	NR	NR	NR	NR	NR	NR	NR	NR	NR	NR	237	Low
Ogunro, 2007	2007	Nigeria	Case-control (group of pregnant women)	Single-center	Urban	Antenatal care	Prospective	2005-2006	Consecutive	NR	NR	NR	NR	NR	NR	NR	NR	NR	NR	NR	NR	NR	NR	9.2	9.2	NR	NR	0	NR	NR	NR	272	Moderate
Okeke, 2012	2012	Nigeria	Cross-sectional	Single-center	Urban, Rural	PMTCT	Retrospective	2007-2009	Consecutive	NR	22-43	NR	100	80.3	37.6	17.5	8.2	16.7	NR	NR	NR	NR	NR	NR	NR	NR	NR	NR	NR	NR	NR	401	Moderate
Okoth, 2006	2006	Kenya	Cross-sectional	Multi-center	Urban, Rural	Antenatal care	Prospective	2001-2002	Consecutive	25.2	12-43	NR	NR	NR	NR	NR	NR	NR	NR	NR	NR	NR	NR	NR	NR	NR	NR	NR	NR	NR	NR	2214	Low
Okusanya, 2013	2013	Nigeria	Cross-sectional	Single-center	Urban, Rural	Antenatal care	Prospective	2010	Consecutive	28.93	20-44	NR	NR	88.8	0.0	100	NR	NR	NR	NR	NR	NR	30.7	NR	NR	NR	NR	NR	NR	NR	NR	205	Low

Study	Year of publication	Country	Design	N Centers	Area	Setting	Timing of data collection	Period of data collection	Sampling	Mean or median age, years	Age limit, years	%HBV Vaccination	%HIV-infected	%Secondary level of education or higher	%Single	%Married	%Divorced	%Widow	%History of transfusion	%Residing in Rural	%History of multiple sexual partners	%Histroty of Surgery	%Primigravida	%Scarification	%Tatoos	%Piercing	%History of circumcision	%History of abortion	%Dental procedure	%Traditional birth attendance	%Polygamous	Sample	Risk of bias
Oladele, 2014	2014	Nigeria	Cross-sectional	Single-center	Urban	Antenatal care	Prospective	2013	Consecutive	30.1	15-49	8.2	5.43	98.9	NR	NR	NR	NR	6.58	NR	7.65	15.3	NR	14.75	2.73	NR	27.27	NR	NR	NR	NR	183	Low
Olaleye, 2013	2013	Nigeria	Baseline data of a Cohort study	Single-center	Urban	Antenatal care	Prospective	2011	Consecutive	NR	NR	0	NR	92	NR	NR	NR	NR	9.3	NR	54	NR	56	53.5	11.6	NR	NR	NR	NR	NR	NR	712	Low
Olokoba, 2011	2011	Nigeria	Cross-sectional	Single-center	Urban, Rural	Antenatal care	Prospective	2008	Consecutive	27.8	17-44	NR	NR	82.2	NR	NR	NR	NR	6.5	NR	NR	15.6	NR	NR	NR	NR	NR	NR	NR	NR	NR	231	Low
Oluboyo, 2014	2014	Nigeria	Cross-sectional	Single-center	Urban, Rural	Antenatal care	Prospective	NR	Consecutive	NR	NR	NR	NR	NR	NR	NR	NR	NR	13	NR	NR	31	NR	NR	NR	NR	NR	NR	NR	NR	NR	100	Moderate
Omolola, 2015	2015	Nigeria	Cross-sectional	Single-center	Urban	Antenatal care	Prospective	2013-2014	Consecutive	29.2	NR	NR	NR	88.2	3	96.8	NR	0.3	12.5	NR	23.2	22.5	30	NR	46.8	NR	NR	NR	NR	NR	8	400	Low
Onah, 2007	2007	Nigeria	Case-control (group of pregnant women)	Single-center	Urban, Rural	Other hospital based (not antenatal care), PMTCT (preventing mother to child transmission of HIV) clinic	Retrospective	2002-2004	Consecutive	NR	15-40	NR	38	89	0	100	0	0	NR	NR	NR	NR	NR	NR	NR	NR	NR	2	NR	NR	NR	162	Moderate
Onakewhor, 2001	2001	Nigeria	Cross-sectional	Single-center	Urban, Rural	Other hospital based (not antenatal care)	Prospective	1997-1998	Random	NR	NR	NR	NR	NR	NR	NR	NR	NR	NR	NR	NR	NR	NR	NR	NR	NR	NR	NR	NR	NR	NR	320	Low
Onakewhor, 2009	2009	Nigeria	Cross-sectional	Single-center	Urban	Antenatal care	Prospective	2005	Consecutive	30.3	NR	NR	8.3	NR	NR	NR	NR	NR	NR	NR	NR	NR	NR	NR	NR	NR	NR	NR	NR	NR	NR	269	Low
Oneh Aba, 2014	2014	Nigeria	Cross-sectional	Multi-center	Urban	Antenatal care	Prospective	2011	Consecutive	NR	16-50	NR	NR	75.62	0	99.75	0	0.25	4.13	NR	37.88	8.75	20.5	29.25	NR	NR	NR	NR	NR	NR	18.88	800	Low
Onwere, 2012	2012	Nigeria	Cross-sectional	Multi-center	Urban, Rural	Antenatal care	Prospective	2010	Consecutive	26.5	NR	NR	0.9	99.1	NR	NR	NR	NR	NR	NR	NR	NR	NR	NR	NR	NR	NR	NR	NR	NR	NR	810	Low
Onwuakor, 2014	2014	Nigeria	Cross-sectional	Single-center	Urban, Rural	Antenatal care	Prospective	2013	Systematic	NR	17-46	NR	NR	96.6	4.3	95.7	0	0	8.0	NR	2.29	NR	64.57	NR	9.7	NR	NR	NR	16	NR	NR	350	Low
Opaleye, 2016	2016	Nigeria	Cross-sectional	Single-center	Urban	Antenatal care	Prospective	NR	Random	NR	15-49	NR	4.9	98.9	4.4	95.6	NR	NR	6.59	NR	NR	NR	NR	14.8	2.7	NR	14.83	NR	NR	NR	NR	182	Low
Osazuwa, 2012	2012	Nigeria	Cross-sectional	Single-center	Rural	Other hospital based (not antenatal care)	Prospective	2010-2011	Consecutive	NR	NR	NR	5.8	NR	NR	NR	NR	NR	NR	NR	NR	NR	68.4	NR	NR	NR	NR	NR	NR	NR	NR	395	Low
Ose Ugbebor, 2011	2011	Nigeria	Cross-sectional	Single-center	Rural	Antenatal care	Prospective	2009-2010	Consecutive	27.3	NR	NR	NR	48.6	NR	NR	NR	NR	NR	NR	NR	NR	55.7	NR	NR	NR	NR	NR	NR	NR	NR	5760	Low

Study	Year of publication	Country	Design	N Centers	Area	Setting	Timing of data collection	Period of data collection	Sampling	Mean or median age, years	Age limit, years	%HBV Vaccination	%HIV-infected	%Secondary level of education or higher	%Single	%Married	%Divorced	%Widow	%History of transfusion	%Residing in Rural	%History of multiple sexual partners	%Histroty of Surgery	%Primigravida	%Scarification	%Tatoos	%Piercing	%History of circumcision	%History of abortion	%Dental procedure	%Traditional birth attendance	%Polygamous	Sample	Risk of bias
Oti, 2018	2018	Nigeria	Cross-sectional	Single-center	Urban, Rural	Antenatal care	Prospective	2016	Consecutive	NR	NR	NR	NR	60	NR	100	0	0	23	NR	NR	NR	51	20.5	70.5	NR	NR	NR	36	NR	56	200	Low
Ouermi, 2009	2009	Burkina Faso	Case-control (group of pregnant women)	Single-center	Urban, Rural	Antenatal care	Prospective	2009	Consecutive	27.65	19-42	NR	50	NR	NR	NR	NR	NR	NR	NR	NR	NR	NR	NR	NR	NR	NR	NR	NR	NR	NR	276	Low
Oyinloye, 2016	2016	Nigeria	Cross-sectional	Single-center	Urban, Rural	Other hospital based (not antenatal care)	Prospective	NR	Consecutive	NR	17-46	NR	NR	NR	NR	NR	NR	NR	NR	NR	NR	NR	NR	NR	NR	NR	NR	NR	NR	NR	NR	91	Moderate
Pascale, 2014	2014	Benin	Cross-sectional	Single-center	Rural	Other hospital based (not antenatal care)	Prospective	2011	Consecutive	26.2	15-41	NR	3.2	NR	4.1	95.9	0	0	NR	NR	NR	NR	19	NR	NR	NR	NR	NR	NR	NR	25.9	283	Low
Pennap, 2011	2011	Nigeria	Cross-sectional	Single-center	Urban, Rural	Antenatal care	Prospective	2009	Consecutive	NR	18-44	NR	NR	NR	7.07	90.76	NR	NR	26.11	NR	NR	13.89	51.67	9.44	NR	NR	NR	NR	NR	NR	NR	180	Low
Pirillo, 2007	2007	Uganda, Rwanda	Cross-sectional	Multi-center	Urban, Rural	Antenatal care, PMTCT	Retrospective	2001-2004	Consecutive	NR	NR	NR	NR	NR	NR	NR	NR	NR	NR	NR	NR	NR	NR	NR	NR	NR	NR	NR	NR	NR	NR	246	Moderate
Pirillo, 2015	2015	Malawi	Cross-sectional	Single-center	Urban	Other hospital based (not antenatal care)	Prospective	2008-2011	Consecutive	27.00	NR	NR	100	NR	NR	NR	NR	NR	NR	NR	NR	NR	34.3	NR	NR	NR	NR	NR	NR	NR	NR	309	Low
Rabiu, 2010	2010	Nigeria	Case-control (group of pregnant women)	Single-center	Urban, Rural	Antenatal care	Retrospective	2006-2007	Systematic	28.00	15-	NR	5.33	77.05	4.10	95.9	0	0	9.84	NR	52.87	NR	51.64	33.61	9.43	NR	20.08	49.18	16.39	NR	13.11	1052	Low
Ramos, 2011	2011	Ethiopia	Cross-sectional	Single-center	Rural	Antenatal care	Prospective	2008	Consecutive	26.1	13-31	NR	1.8	NR	NR	NR	NR	NR	NR	100	NR	NR	NR	NR	NR	NR	NR	NR	NR	NR	NR	165	Low
Randriamahazo, 2015	2015	Madagascar	Cross-sectional	Single-center	Urban	Antenatal care	Prospective	2012	Consecutive	26.5	14-44	0.4	NR	NR	NR	NR	NR	NR	NR	0	NR	NR	NR	NR	NR	NR	NR	NR	NR	NR	NR	1050	Low
Rashid, 2014	2014	Tanzania	Cross-sectional	Single-center	Urban, Rural	Antenatal care	Prospective	2010	Consecutive	28.5	15-	NR	9.7	48	7.1	92.6	0.3	0	11.6	NR	38.4	NR	31	NR	NR	NR	NR	NR	NR	NR	NR	310	Low
Rezk, 2017	2017	Egypt	Case-control (group of pregnant women)	Single-center	Urban	Antenatal care	Prospective	2012-2017	Consecutive	NR	NR	NR	NR	NR	NR	NR	NR	NR	6.4	NR	NR	NR	56.7	NR	NR	NR	NR	NR	NR	NR	NR	54872	Low

Study	Year of publication	Country	Design	N Centers	Area	Setting	Timing of data collection	Period of data collection	Sampling	Mean or median age, years	Age limit, years	%HBV Vaccination	%HIV-infected	%Secondary level of education or higher	%Single	%Married	%Divorced	%Widow	%History of transfusion	%Residing in Rural	%History of multiple sexual partners	%Histroty of Surgery	%Primigravida	%Scarification	%Tatoos	%Piercing	%History of circumcision	%History of abortion	%Dental procedure	%Traditional birth attendance	%Polygamous	Sample	Risk of bias
Saleh, 2008	2008	Togo	Cross-sectional	Single-center	Urban, Rural	Other hospital based (not antenatal care)	Prospective	2007	Consecutive	NR	NR	NR	NR	NR	NR	NR	NR	NR	NR	NR	NR	NR	NR	NR	NR	NR	NR	NR	NR	NR	NR	20	Low
Saleh, 2008	2008	Egypt	Cross-sectional	Multi-center	Rural	Antenatal care	Prospective	1997-2006	Consecutive	NR	NR	NR	NR	NR	NR	NR	NR	NR	NR	100	NR	NR	NR	NR	NR	NR	NR	NR	NR	NR	NR	2171	Low
Sangaré, 2009	2009	Burkina Faso	Cross-sectional	Single-center	Urban	Antenatal care	Prospective	2005	Consecutive	24.6	15-43	NR	5.56	28.89	34.44	59.3	0.28	NR	4.72	NR	NR	13.89	NR	NR	NR	NR	30.28	NR	13.61	NR	NR	360	Low
Sangaré, 2011	2011	Burkina Faso	Cross-sectional	Multi-center	Urban, Rural	Antenatal care, Other hospital based (not antenatal care)	Prospective	2006-2007	Consecutive	24.8	15-45	NR	NR	NR	NR	NR	NR	NR	NR	NR	NR	NR	NR	NR	NR	NR	NR	NR	NR	NR	NR	1139	Low
Sbiti, 2016	2016	Morocco	Cross-sectional	Single-center	Urban	Antenatal care	Prospective	2014-2015	Consecutive	28.00	17-43	2.4	NR	NR	NR	NR	NR	NR	NR	NR	NR	NR	43.8	NR	NR	NR	NR	NR	NR	NR	NR	1021	Moderate
Serme, 2005	2005	Burkina Faso	Cross-sectional	Multi-center	Urban, Rural	Other hospital based (not antenatal care)	Prospective	2002	Consecutive	27.2	16-43	NR	7.5	NR	15.5	84	NR	0.5	23.5	NR	NR	9	NR	NR	NR	NR	58	NR	18.5	NR	NR	200	Low
Shelb, 2009	2009	Egypt	Cross-sectional	Multi-center	Rural	Antenatal care	Prospective	1997-2001	Consecutive	NR	NR	NR	NR	NR	NR	NR	NR	NR	NR	NR	NR	NR	NR	NR	NR	NR	NR	NR	NR	NR	NR	1863	Moderate
Sidibé, 2001	2001	Mali	Cross-sectional	Multi-center	Urban, Rural	Antenatal care	Prospective	1994-1999	Consecutive	34.1	17-46	NR	NR	NR	NR	NR	NR	NR	NR	NR	NR	NR	NR	65.1	NR	NR	NR	NR	NR	NR	NR	829	Low
Simpore, 2005	2005	Burkina Faso	Cross-sectional	Single-center	Urban	Antenatal care	Prospective	2001-2002	Random	25.9	18-44	NR	10.6	NR	0.7	99.3	0	0	45	NR	NR	NR	28.7	NR	NR	NR	NR	NR	NR	NR	NR	547	Low
Simpore, 2006	2006	Burkina Faso	Cross-sectional	Single-center	Urban	Other hospital based (not antenatal care)	Prospective	2004-2005	Random	25.92	18-45	NR	61.61	NR	NR	NR	NR	NR	NR	NR	NR	NR	NR	NR	NR	NR	NR	NR	NR	NR	NR	336	Moderate
Simpore, 2006	2006	Burkina Faso	Cross-sectional	Single-center	Urban	Antenatal care	Prospective	2003-2005	Random	25.92	18-44	NR	25.17	NR	NR	NR	NR	NR	NR	NR	NR	NR	NR	NR	NR	NR	NR	NR	NR	NR	NR	429	Low
Stoszek, 2006	2006	Egypt	Baseline data of a Cohort study	Multi-center	Urban	Antenatal care	Prospective	1997-2003	Consecutive	24.00	16-48	NR	NR	NR	NR	NR	NR	NR	2.4	NR	NR	26.6	NR	NR	NR	NR	NR	NR	NR	NR	NR	2587	Low
Strand, 2003	2003	Angola	Case-control (group of pregnant women)	Multi-center	Urban, Rural	Antenatal care	Prospective	1999	Consecutive	23.3	15-38	NR	5	NR	NR	NR	NR	NR	NR	NR	NR	NR	NR	NR	NR	NR	NR	NR	NR	NR	NR	60	Low
Tegene, 2014	2014	Ethiopia	Cross-sectional	Multi-center	Urban	Antenatal care	Prospective	2012	Consecutive	25.8	NR	NR	NR	8.5	8.3	91.3	0.4	0.0	4.53	NR	14.76	9.06	60.0	NR	21.5	NR	NR	16.6	12.08	75.09	NR	265	Low

Study	Year of publication	Country	Design	N Centers	Area	Setting	Timing of data collection	Period of data collection	Sampling	Mean or median age, years	Age limit, years	%HBV Vaccination	%HIV-infected	%Secondary level of education or higher	%Single	%Married	%Divorced	%Widow	%History of transfusion	%Residing in Rural	%History of multiple sexual partners	%Histroty of Surgery	%Primigravida	%Scarification	%Tatoos	%Piercing	%History of circumcision	%History of abortion	%Dental procedure	%Traditional birth attendance	%Polygamous	Sample	Risk of bias
Thumbiran, 2014	2014	South Africa	Cross-sectional	Single-center	Rural	Antenatal care	Retrospective	2009	Systematic	23.00	16-47	NR	41.6	NR	NR	NR	NR	NR	NR	NR	NR	NR	NR	NR	NR	NR	NR	NR	NR	NR	NR	517	Low
Umare, 2016	2016	Ethiopia	Cross-sectional	Single-center	Urban, Rural	Antenatal care	Prospective	2015	Consecutive	25.00	15-40	NR	0.0	21.1	1.6	94	2.2	2.2	0.63	56.9	13.8	4.7	25.8	NR	9.75	6.9	40.88	23.6	12.9	50	NR	318	Low
Usanga, 2011	2011	Nigeria	Cross-sectional	Single-center	Urban, Rural	Antenatal care	Prospective	2012-2013	Consecutive	NR	15-49	NR	6.8	68.8	NR	NR	NR	NR	NR	NR	NR	NR	NR	NR	NR	NR	NR	NR	NR	NR	NR	562	Low
Utoo, 2011	2011	Nigeria	Cross-sectional	Single-center	Urban, Rural	Other hospital based (not antenatal care)	Prospective	2012	Consecutive	25.5	-35	NR	4.55	75	NR	NR	NR	NR	NR	NR	NR	NR	8.3	NR	NR	NR	NR	NR	NR	NR	NR	836	Low
Utoo, 2013	2013	Nigeria	Cross-sectional	Single-center	Urban, Rural	Antenatal care	Prospective	2010	Consecutive	26.9	18-38	NR	NR	63.6	NR	NR	NR	NR	NR	NR	NR	NR	9.1	NR	NR	NR	NR	NR	NR	NR	NR	836	Low
Volker, 2017	2017	Ghana	Cross-sectional	Single-center	Rural	Other hospital based (not antenatal care)	Retrospective	2011-2012	Consecutive	26.3	14-48	NR	0.6	NR	NR	NR	NR	NR	NR	NR	NR	NR	NR	NR	NR	NR	NR	NR	NR	NR	NR	174	Low
Wurie, 2005	2005	Sierra Leone	Cross-sectional	Single-center	Urban	Antenatal care	Prospective	NR	Consecutive	28.00	16-40	NR	NR	100	NR	NR	NR	NR	NR	NR	NR	NR	6.2	NR	NR	NR	NR	NR	NR	NR	NR	302	Moderate
Ya'Aba, 2009	2009	Nigeria	Cross-sectional	Multi-center	Urban, Rural	Antenatal care	Prospective	2005-2006	Consecutive	27.5	15-40	NR	16.9	NR	NR	NR	NR	NR	NR	NR	NR	NR	NR	NR	NR	NR	NR	NR	NR	NR	NR	203	High
Yakasai, 2012	2012	Nigeria	Cross-sectional	Single-center	Urban, Rural	Antenatal care	Prospective	2011	Random	27.6	18-49	NR	NR	76.2	0.0	98.2	2.0	0.0	15.0	NR	NR	7.9	NR	NR	15.1	7.9	NR	17.6	5.1	NR	NR	303	Low
Yohanes, 2016	2016	Ethiopia	Cross-sectional	Single-center	Urban, Rural	Antenatal care	Prospective	2015	Consecutive	26.0	15-39	NR	NR	54.4	2.6	96.6	0.0	0.9	6.9	7.8	5.2	8.0	3.6	NR	6.3	4.2	NR	22.7	22.3	8.0	NR	232	Low
Zahran, 2010	2010	Egypt	Cross-sectional	Single-center	Urban, Rural	Antenatal care	Prospective	2008-2009	Consecutive	23.00	NR	NR	NR	NR	NR	NR	NR	NR	14	78.2	NR	14.2	NR	NR	NR	NR	NR	NR	NR	NR	NR	500	Low
Zeba,2011	2011	Burkina Faso	Cross-sectional	Single-center	Urban	Antenatal care, PMTCT	Prospective	2009	Consecutive	28.3	16-45	NR	62.27	15.15	NR	NR	NR	NR	NR	NR	NR	NR	NR	15.32	11.7	NR	42.67	NR	NR	NR	NR	607	Low
Zenebe, 2015	2015	Ethiopia	Cross-sectional	Multi-center	Urban, Rural	Antenatal care	Prospective	2013	Systematic	25.7	25-30	NR	NR	1.8	NR	93.1	1.8	0.3	NR	32.7	NR	NR	NR	NR	NR	NR	NR	NR	NR	NR	NR	318	Low
Mutagoma, 2017	2017	Rwanda	Cross-sectional	Multi-center	Urban, Rural	Antenatal care	Prospective	2011	Consecutive	NR	15-49	NR	NR	11.2	12.7	87.3	NR	NR	NR	49.9	NR	NR	NR	NR	NR	NR	NR	NR	NR	NR	NR	13121	Low
Zenebe, 2014	2014	Ethiopia	Cross-sectional	Multi-center	Urban, Rural	Antenatal care	Prospective	2013	Consecutive	25.7	NR	NR	6.6	35.8	4.7	93.1	A.9	0.3	NR	NR	NR	NR	NR	NR	NR	NR	NR	NR	NR	NR	NR	318	Low

Supplemental Table 5 : ***Sources of heterogeneity of the prevalence of HBV infection in pregnant women in Africa***

	Univariable model			Multivariable final model*	
	**P value**	**Coefficient (95%CI)**	**R^2**	**P value**	**Adjusted coefficient (95%CI)**
Region (Eastern)	< 0.0001		13.8		
- Middle		0.0797 (0.0216 ; 0.1379)		0.0054	0.0943 (0.0278; 0.1608)
- Northern		-0.0713 (-0.1279 ; -0.0146)		0.6135	-0.0295 (-0.1439; 0.0849)
- Southern		-0.0407 (-0.1063 ; 0.0249)		0.9391	0.0039 (-0.0958; 0.1035)
- Western		0.0553 (0.0179 ; 0.0926)		0.0072	0.0937 (0.0254; 0.1620)
Area (Rural)	< 0.0001		24.8		
- Urban		-0.1087 (-0.1602 ; -0.0572)		0.0001	-0.0853 (-0.1288; -0.0419)
- Both		-0.1000 (-0.1458 ; -0.0542)		< 0.0001	-0.0808 (-0.1205; -0.0410)
Year	0.4207		0.0		
GDI	0.0003	-0.6401 (-0.9856 ; -0.2946)	6.8	0.0278	-0.8913 (-1.6854; -0.0972)
Life expectancy at birth, female	0.0001	-0.0043 (-0.0065 ; -0.0021)	8.3	0.3430	0.0022 (-0.0023; 0.0066)
Life expectancy at birth, male	0.0014		5.9		
Gross national income per capita, female	0.0043	-0.0298 (-0.0502 ; -0.0094)	0.6	0.2559	0.0000 (-0.0000; 0.0000)
Gross national income per capita, male	< 0.0001	-0.0194 (-0.0343 ; -0.0044)	1.9	0.0664	-0.0000 (-0.0000; 0.0000)
Expected years of schooling, female	< 0.0001	-0.0156 (-0.0224 ; -0.0088)	19.7	0.0259	-0.0425 (-0.0051; -0.0800)
Expected years of schooling, male	0.0065	-0.0131 (-0.0225 ; -0.0037)	5.1	0.0722	0.0320 (-0.0670; 0.0029)
Gender inequality index[¥]	< 0.0001	0.4026 (0.2545 ; 0.5507)	30.6		
Human development index, female	< 0.0001	-0.4127 (-0.5874 ; 0.2380)	16.6		
Human development index, male	< 0.0001	-0.3844 (-0.5752 ; -0.1935)	8.0		
Mean years of schooling, female	0.0005	-0.0124 (-0.0194 ; -0.0055)	8.6	0.1132	0.0181 (-0.0043; 0.0406)
Mean years of schooling, male	0.0175	-0.0093 (-0.0169 ; -0.0016)	1.3		
Population with at least some secondary education, female	0.0336	-0.0009 (-0.0018 ; -0.0001)	0.0		
Population with at least some secondary education, male	0.1953	-0.0005 (-0.0013 ; 0.0003)	0.1	0.0243	-0.0022 (-0.0041; -0.0003)
Share of seats in parliament (%held by women)	0.0088	-0.0015 (-0.0026 ; -0.0004)	1.2	0.2428	-0.0014 (-0.0038; 0.0010)
Unemployment rate, female to male ratio	0.0007	-0.0485 (-0.0764 ; -0.0205)	2.1		
Single center study	0.786	0.0046 (-0.0287 ; 0.0380)	0.0		
Sample size	0.0136	-0.0179 (-0.0322 ; -0.0037)	3.2	0.0786	-0.0117 (-0.0247; 0.0013)

* N studies = 109; Residual heterogeneity: I^2 = 88.9%; Explained heterogeneity: R^2 = 53.8%

[¥] Not included in multivariable model because of missing data (38.5%)

Supplemental Table 6 : ***Sources of heterogeneity of the prevalence of HCV infection in pregnant women in Africa***

	**Univariable analysis**			**Multivariable analysis***	
	**P value**	**Coefficient (95%CI)**	**R^2**	**P value**	**Adjusted coefficient (95%CI)**
Region (Eastern)	0.5083		0.0		
- Middle					
- Northern					
- Southern					
- Western					
Area (Rural)	0.5035		0.0		
- Urban					
- Both					
Year	0.0571	-0.0046 (-0.0094 ; 0.0001)	11.1	0.0757	-0.0050 (-0.0104; 0.0005)
GDI	0.3923		0.0		
Life expectancy at birth, female	0.2868		0.0		
Life expectancy at birth, male	0.2916		0.0		
Gross national income per capita, female	0.8293		0.0		
Gross national income per capita, male	0.4443		0.0		
Expected years of schooling, female	0.2486	0.0069 (-0.0048 ; 0.0185)	0.0	0.3662	0.0163 (-0.0191; 0.0518)
Expected years of schooling, male	0.1766	0.0098 (-0.0044 ; 0.0239)	0.0	0.8176	-0.0051 (-0.0485; 0.0383)
Gender inequality index[¥]	0.0531		0.0		
Human development index, female	0.2749		0.0		
Human development index, male	0.3283		0.0		
Mean years of schooling, female	0.3518		0.0		
Mean years of schooling, female	0.6797		0.0		
Population with at least some secondary education, female	0.2330	0.0009 (-0.0006 ; 0.0024)	0.0	0.5936	-0.0015 (-0.0071; 0.0041)
Population with at least some secondary education, male	0.2296	0.0008 (-0.0005 ; 0.0020)	0.0	0.8796	0.0003 (-0.0041; 0.0048)
Share of seats in parliament (%held by women)	0.0210	-0.0024 (-0.0045 ; -0.0004)	0.1	0.0339	-0.0029 (-0.0056; -0.0002)
Unemployment rate, female to male ratio	0.2452	0.0183 (-0.0126 ; 0.0492)	0.0	0.4208	0.0173 (-0.0248; 0.0595)
Single center study	0.6118		0.5		
Sample size	0.2038	-0.0108 (-0.0275 ; 0.0059)	15.5	0.0922	-0.0182 (-0.0394; 0.0030)

*N studies: 57; Residual heterogeneity: I^2 = 96.5%; Explained heterogeneity: R = 0.95%

Supplemental Figure 1 : Meta-analysis results for HBV infection prevalence among pregnant women in Africa, by country

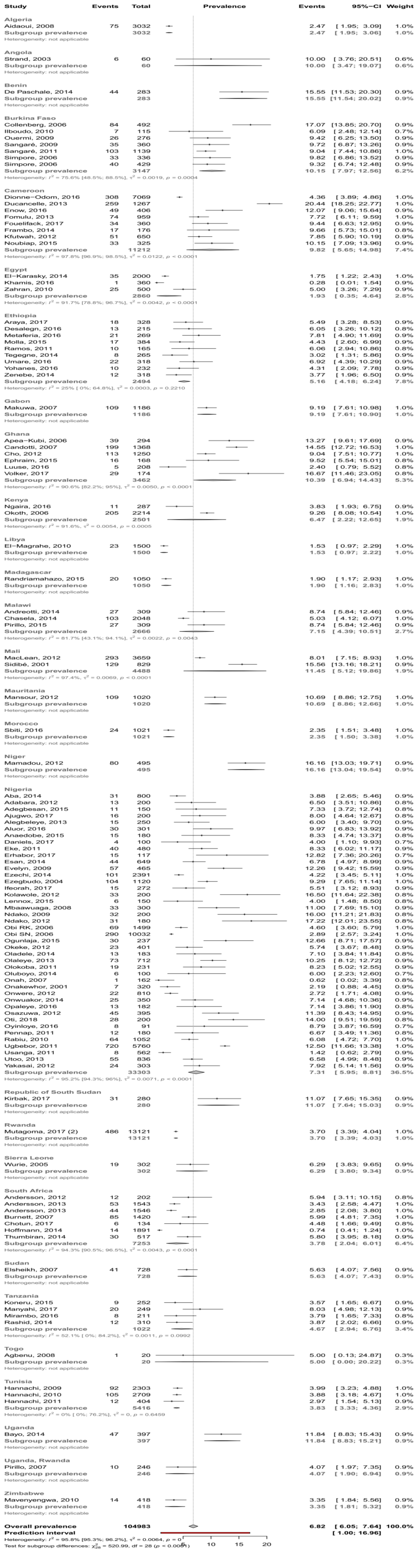

Supplemental Figure 2 : Meta-analysis results for HCV infection prevalence among pregnant women in Africa, by country

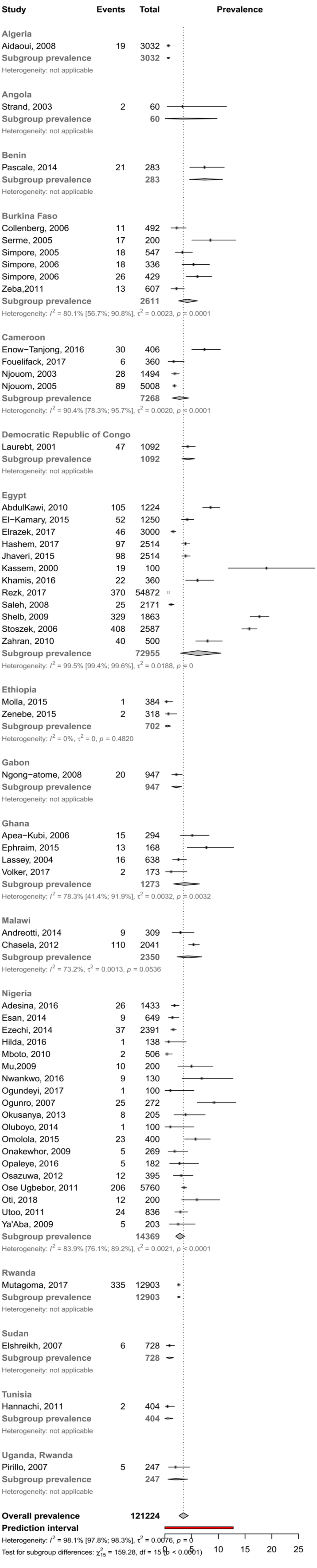

Study	Events	Total	Events	95%−CI	Weight
**Algeria**					
Aidaoui, 2008	19	3032	0.63	[0.38; 0.98]	1.9%
**Subgroup prevalence**		**3032**	**0.63**	**[0.37; 0.94]**	**1.9%**
Heterogeneity: not applicable					
**Angola**					
Strand, 2003	2	60	3.33	[0.41; 11.53]	1.2%
**Subgroup prevalence**		**60**	**3.33**	**[0.05; 9.78]**	**1.2%**
Heterogeneity: not applicable					
**Benin**					
Pascale, 2014	21	283	7.42	[4.65; 11.12]	1.7%
**Subgroup prevalence**		**283**	**7.42**	**[4.63; 10.79]**	**1.7%**
Heterogeneity: not applicable					
**Burkina Faso**					
Collenberg, 2006	11	492	2.24	[1.12; 3.97]	1.8%
Serme, 2005	17	200	8.50	[5.03; 13.26]	1.6%
Simpore, 2005	18	547	3.29	[1.96; 5.15]	1.8%
Simpore, 2006	18	336	5.36	[3.21; 8.33]	1.7%
Simpore, 2006	26	429	6.06	[4.00; 8.75]	1.7%
Zeba,2011	13	607	2.14	[1.15; 3.63]	1.8%
**Subgroup prevalence**		**2611**	**4.16**	**[2.56; 6.10]**	**10.4%**
Heterogeneity: I^2 = 80.1% [56.7%; 90.8%], τ^2 = 0.0023, p = 0.0001					
**Cameroon**					
Enow−Tanjong, 2016	30	406	7.39	[5.04; 10.38]	1.7%
Fouelifack, 2017	6	360	1.67	[0.61; 3.59]	1.7%
Njouom, 2003	28	1494	1.87	[1.25; 2.70]	1.8%
Njouom, 2005	89	5008	1.78	[1.43; 2.18]	1.9%
**Subgroup prevalence**		**7268**	**2.71**	**[1.36; 4.50]**	**7.2%**
Heterogeneity: I^2 = 90.4% [78.3%; 95.7%], τ^2 = 0.0020, p < 0.0001					
**Democratic Republic of Congo**					
Laurebt, 2001	47	1092	4.30	[3.18; 5.68]	1.8%
**Subgroup prevalence**		**1092**	**4.30**	**[3.18; 5.59]**	**1.8%**
Heterogeneity: not applicable					
**Egypt**					
AbdulKawi, 2010	105	1224	8.58	[7.07; 10.29]	1.8%
El−Kamary, 2015	52	1250	4.16	[3.12; 5.42]	1.8%
Elrazek, 2017	46	3000	1.53	[1.12; 2.04]	1.9%
Hashem, 2017	97	2514	3.86	[3.14; 4.69]	1.9%
Jhaveri, 2015	98	2514	3.90	[3.18; 4.73]	1.9%
Kassem, 2000	19	100	19.00	[11.84; 28.07]	1.4%
Khamis, 2016	22	360	6.11	[3.87; 9.11]	1.7%
Rezk, 2017	370	54872	0.67	[0.61; 0.75]	1.9%
Saleh, 2008	25	2171	1.15	[0.75; 1.70]	1.9%
Shelb, 2009	329	1863	17.66	[15.95; 19.47]	1.8%
Stoszek, 2006	408	2587	15.77	[14.39; 17.23]	1.9%
Zahran, 2010	40	500	8.00	[5.78; 10.73]	1.8%
**Subgroup prevalence**		**72955**	**6.17**	**[2.93; 10.49]**	**21.6%**
Heterogeneity: I^2 = 99.5% [99.4%; 99.6%], τ^2 = 0.0188, p = 0					
**Ethiopia**					
Molla, 2015	1	384	0.26	[0.01; 1.44]	1.7%
Zenebe, 2015	2	318	0.63	[0.08; 2.25]	1.7%
**Subgroup prevalence**		**702**	**0.41**	**[0.03; 1.08]**	**3.4%**
Heterogeneity: I^2 = 0%, τ^2 = 0, p = 0.4820					
**Gabon**					
Ngong−atome, 2008	20	947	2.11	[1.29; 3.24]	1.8%
**Subgroup prevalence**		**947**	**2.11**	**[1.28; 3.14]**	**1.8%**
Heterogeneity: not applicable					
**Ghana**					
Apea−Kubi, 2006	15	294	5.10	[2.88; 8.28]	1.7%
Ephraim, 2015	13	168	7.74	[4.18; 12.87]	1.6%
Lassey, 2004	16	638	2.51	[1.44; 4.04]	1.8%
Volker, 2017	2	173	1.16	[0.14; 4.11]	1.6%
**Subgroup prevalence**		**1273**	**3.68**	**[1.60; 6.51]**	**6.6%**
Heterogeneity: I^2 = 78.3% [41.4%; 91.9%], τ^2 = 0.0032, p = 0.0032					
**Malawi**					
Andreotti, 2014	9	309	2.91	[1.34; 5.46]	1.7%
Chasela, 2012	110	2041	5.39	[4.45; 6.46]	1.9%
**Subgroup prevalence**		**2350**	**4.31**	**[2.28; 6.92]**	**3.6%**
Heterogeneity: I^2 = 73.2%, τ^2 = 0.0013, p = 0.0536					
**Nigeria**					
Adesina, 2016	26	1433	1.81	[1.19; 2.65]	1.8%
Esan, 2014	9	649	1.39	[0.64; 2.62]	1.8%
Ezechi, 2014	37	2391	1.55	[1.09; 2.13]	1.9%
Hilda, 2016	1	138	0.72	[0.02; 3.97]	1.5%
Mboto, 2010	2	506	0.40	[0.05; 1.42]	1.8%
Mu,2009	10	200	5.00	[2.42; 9.00]	1.6%
Nwankwo, 2016	9	130	6.92	[3.21; 12.74]	1.5%
Ogundeyi, 2017	1	100	1.00	[0.03; 5.45]	1.4%
Ogunro, 2007	25	272	9.19	[6.04; 13.27]	1.7%
Okusanya, 2013	8	205	3.90	[1.70; 7.54]	1.6%
Oluboyo, 2014	1	100	1.00	[0.03; 5.45]	1.4%
Omolola, 2015	23	400	5.75	[3.68; 8.50]	1.7%
Onakewhor, 2009	5	269	1.86	[0.61; 4.28]	1.7%
Opaleye, 2016	5	182	2.75	[0.90; 6.29]	1.6%
Osazuwa, 2012	12	395	3.04	[1.58; 5.25]	1.7%
Ose Ugbebor, 2011	206	5760	3.58	[3.11; 4.09]	1.9%
Oti, 2018	12	200	6.00	[3.14; 10.25]	1.6%
Utoo, 2011	24	836	2.87	[1.85; 4.24]	1.8%
Ya'Aba, 2009	5	203	2.46	[0.80; 5.65]	1.6%
**Subgroup prevalence**		**14369**	**2.80**	**[2.02; 3.69]**	**31.7%**
Heterogeneity: I^2 = 83.9% [76.1%; 89.2%], τ^2 = 0.0021, p < 0.0001					
**Rwanda**					
Mutagoma, 2017	335	12903	2.60	[2.33; 2.89]	1.9%
**Subgroup prevalence**		**12903**	**2.60**	**[2.33; 2.88]**	**1.9%**
Heterogeneity: not applicable					
**Sudan**					
Elshreikh, 2007	6	728	0.82	[0.30; 1.79]	1.8%
**Subgroup prevalence**		**728**	**0.82**	**[0.27; 1.64]**	**1.8%**
Heterogeneity: not applicable					
**Tunisia**					
Hannachi, 2011	2	404	0.50	[0.06; 1.78]	1.7%
**Subgroup prevalence**		**404**	**0.50**	**[0.01; 1.49]**	**1.7%**
Heterogeneity: not applicable					
**Uganda, Rwanda**					
Pirillo, 2007	5	247	2.02	[0.66; 4.66]	1.7%
**Subgroup prevalence**		**247**	**2.02**	**[0.57; 4.23]**	**1.7%**
Heterogeneity: not applicable					
**Overall prevalence**		**121224**	**3.45**	**[2.62; 4.38]**	**100.0%**
**Prediction interval**				**[0.00; 12.79]**	

Heterogeneity: I^2 = 98.1% [97.8%; 98.3%], τ^2 = 0.0076, p = 0

Test for subgroup differences: χ^2_{15} = 159.28, df = 15 (p < 0.0001)

Supplemental Figure 3 : Funnel plot for HBV infection prevalence among pregnant women in Africa

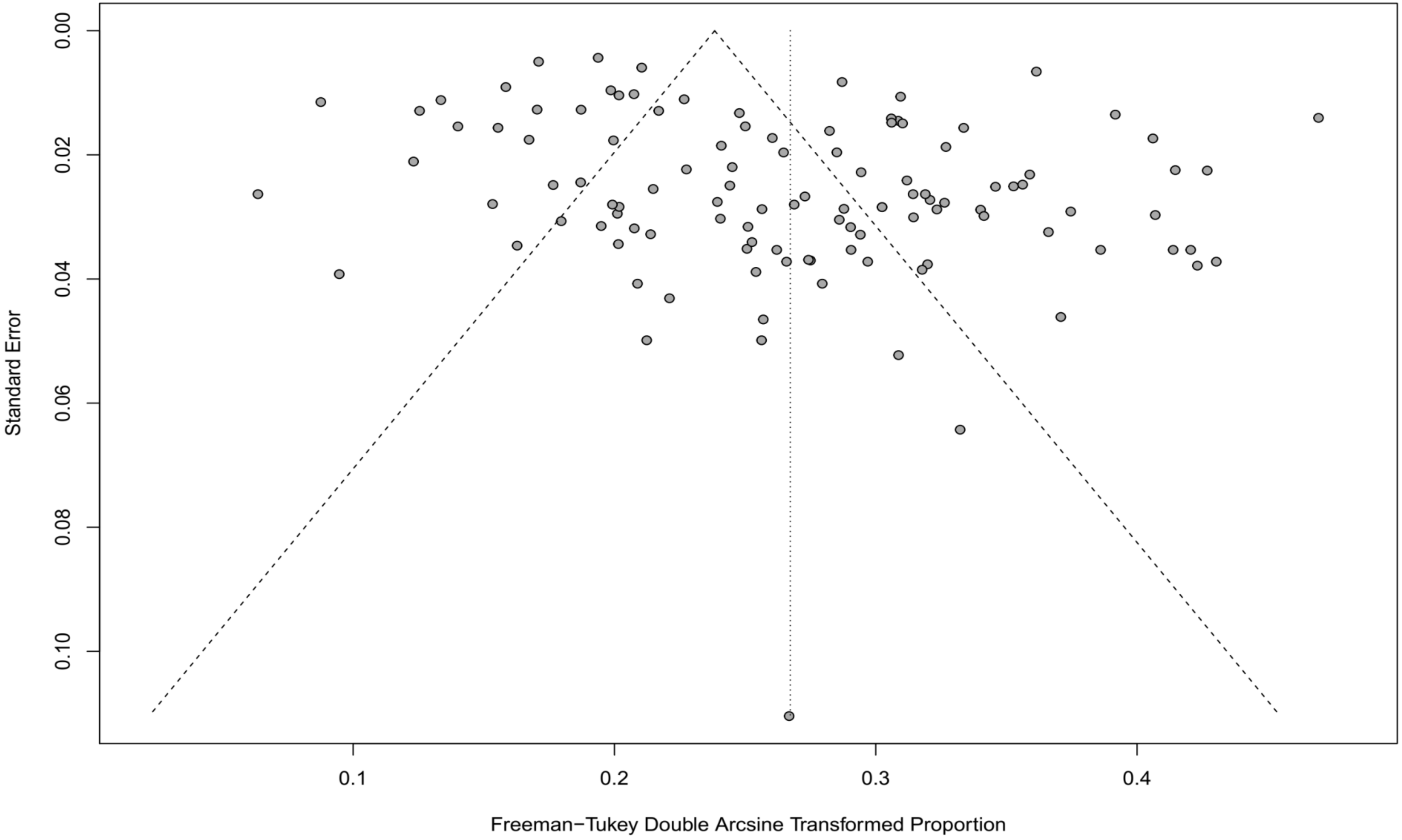

Supplemental Figure 4 : Funnel plot for HCV infection prevalence among pregnant women in Africa

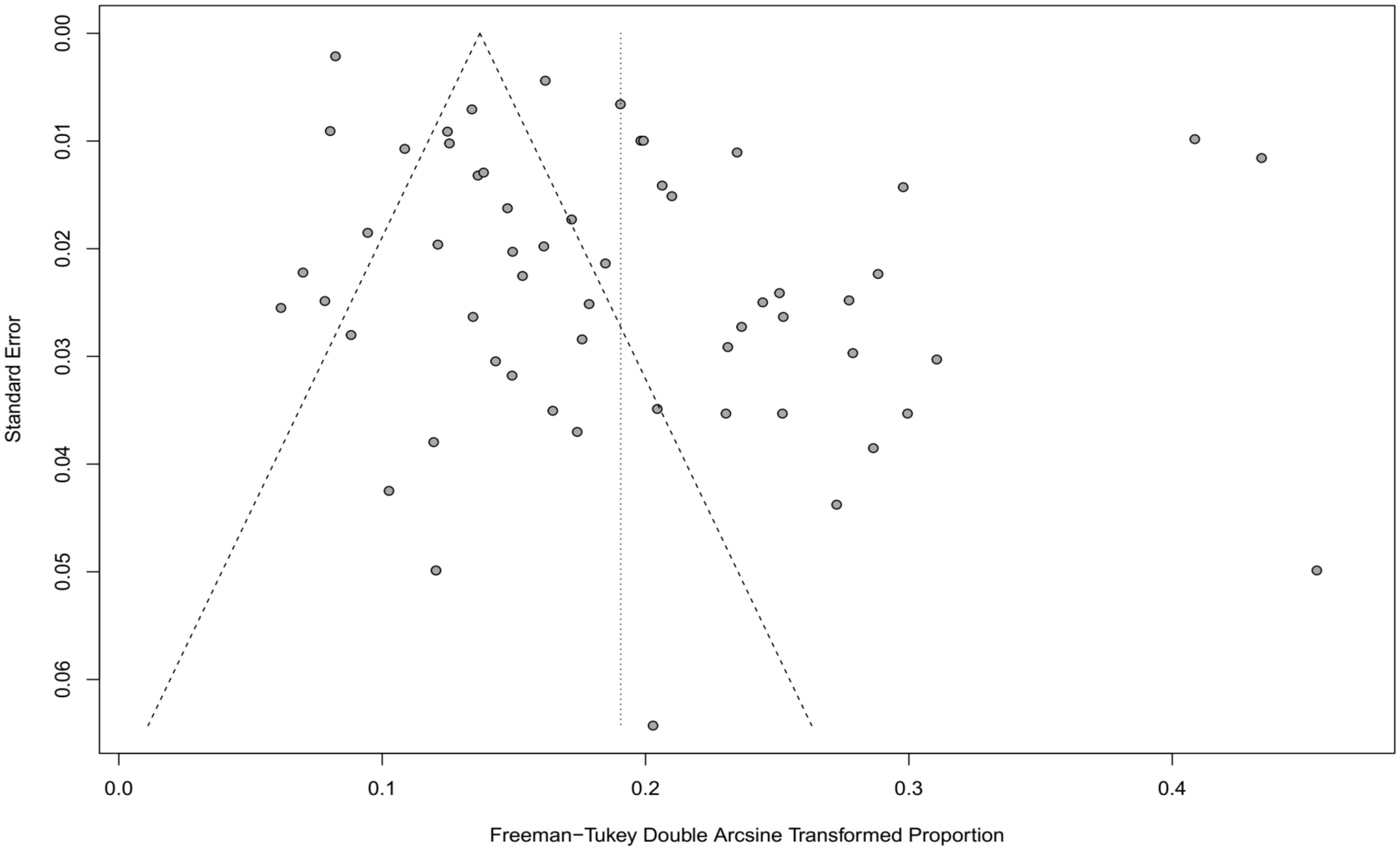

Supplemental Figure 5 : Meta-analysis results for HBV infection prevalence among pregnant women in Africa, by area

Study	Events	Total	Events	95%-CI	Weight
**Rural**					
Bayo, 2014	47	397	11.84	[8.83; 15.43]	0.9%
De Paschale, 2014	44	283	15.55	[11.53; 20.30]	0.9%
Ducancelle, 2013	259	1267	20.44	[18.25; 22.77]	1.0%
Ephraim, 2015	16	168	9.52	[5.54; 15.01]	0.8%
Erhabor, 2017	15	117	12.82	[7.36; 20.26]	0.7%
Kolawole, 2012	33	200	16.50	[11.64; 22.38]	0.8%
Noubiap, 2015	33	325	10.15	[7.09; 13.96]	0.9%
Osazuwa, 2012	45	395	11.39	[8.43; 14.95]	0.9%
Ramos, 2011	10	165	6.06	[2.94; 10.86]	0.8%
Thumbiran, 2014	30	517	5.80	[3.95; 8.18]	0.9%
Ugbebor, 2011	720	5760	12.50	[11.66; 13.38]	1.0%
Volker, 2017	29	174	16.67	[11.46; 23.05]	0.8%
**Subgroup prevalence**		**9768**	**12.18**	**[9.74; 14.85]**	**10.4%**
Heterogeneity: I^2 = 89.4% [83.5%; 93.2%], τ^2 = 0.0038, p < 0.0001					
**Urban**					
Aba, 2014	31	800	3.88	[2.65; 5.46]	0.9%
Adabara, 2012	13	200	6.50	[3.51; 10.86]	0.8%
Adegbesan, 2015	11	150	7.33	[3.72; 12.74]	0.8%
Aidaoui, 2008	75	3032	2.47	[1.95; 3.09]	1.0%
Andersson, 2012	12	202	5.94	[3.11; 10.15]	0.8%
Apea−Kubi, 2006	39	294	13.27	[9.61; 17.69]	0.9%
Evelyn, 2009	57	465	12.26	[9.42; 15.59]	0.9%
Ezechi, 2014	101	2391	4.22	[3.45; 5.11]	1.0%
Fomulu, 2013	74	959	7.72	[6.11; 9.59]	1.0%
Fouelifack, 2017	34	360	9.44	[6.63; 12.95]	0.9%
Hannachi, 2011	12	404	2.97	[1.54; 5.13]	0.9%
Hoffmann, 2014	14	1891	0.74	[0.41; 1.24]	1.0%
Manyahi, 2017	20	249	8.03	[4.98; 12.13]	0.9%
Ngaira, 2016	11	287	3.83	[1.93; 6.75]	0.9%
Oladele, 2014	13	183	7.10	[3.84; 11.84]	0.8%
Olaleye, 2013	73	712	10.25	[8.12; 12.72]	0.9%
Opaleye, 2016	13	182	7.14	[3.86; 11.90]	0.8%
Pirillo, 2015	27	309	8.74	[5.84; 12.46]	0.9%
Randriamahazo, 2015	20	1050	1.90	[1.17; 2.93]	1.0%
Sangaré, 2009	35	360	9.72	[6.87; 13.26]	0.9%
Sbiti, 2016	24	1021	2.35	[1.51; 3.48]	1.0%
Simpore, 2006	33	336	9.82	[6.86; 13.52]	0.9%
Simpore, 2006	40	429	9.32	[6.74; 12.48]	0.9%
Tegegne, 2014	8	265	3.02	[1.31; 5.86]	0.9%
Wurie, 2005	19	302	6.29	[3.83; 9.65]	0.9%
**Subgroup prevalence**		**16833**	**6.01**	**[4.53; 7.69]**	**22.4%**
Heterogeneity: I^2 = 94.2% [92.5%; 95.5%], τ^2 = 0.0063, p < 0.0001					
**Urban, Rural**					
Agbenu, 2008	1	20	5.00	[0.13; 24.87]	0.3%
Ajugwo, 2017	16	200	8.00	[4.64; 12.67]	0.8%
Alegbeleye, 2013	15	250	6.00	[3.40; 9.70]	0.9%
Aluor, 2016	30	301	9.97	[6.83; 13.92]	0.9%
Anaedobe, 2015	15	180	8.33	[4.74; 13.37]	0.8%
Andersson, 2013	53	1543	3.43	[2.58; 4.47]	1.0%
Andersson, 2013	44	1546	2.85	[2.08; 3.80]	1.0%
Andreotti, 2014	27	309	8.74	[5.84; 12.46]	0.9%
Araya, 2017	18	328	5.49	[3.28; 8.53]	0.9%
Burnett, 2007	85	1420	5.99	[4.81; 7.35]	1.0%
Candotti, 2007	199	1368	14.55	[12.72; 16.53]	1.0%
Chasela, 2014	103	2048	5.03	[4.12; 6.07]	1.0%
Cho, 2012	113	1250	9.04	[7.51; 10.77]	1.0%
Chotun, 2017	6	134	4.48	[1.66; 9.49]	0.8%
Collenberg, 2006	84	492	17.07	[13.85; 20.70]	0.9%
Daniels, 2017	4	100	4.00	[1.10; 9.93]	0.7%
Desalegn, 2016	13	215	6.05	[3.26; 10.12]	0.8%
Dionne−Odom, 2016	308	7069	4.36	[3.89; 4.86]	1.0%
Eke, 2011	40	480	8.33	[6.02; 11.17]	0.9%
El−Karasky, 2014	35	2000	1.75	[1.22; 2.43]	1.0%
El−Magrahe, 2010	23	1500	1.53	[0.97; 2.29]	1.0%
Elsheikh, 2007	41	728	5.63	[4.07; 7.56]	0.9%
Enow, 2016	49	406	12.07	[9.06; 15.64]	0.9%
Esan, 2014	44	649	6.78	[4.97; 8.99]	0.9%
Ezegbudo, 2004	104	1120	9.29	[7.65; 11.14]	1.0%
Frambo, 2014	17	176	9.66	[5.73; 15.01]	0.8%
Hannachi, 2009	92	2303	3.99	[3.23; 4.88]	1.0%
Hannachi, 2010	105	2709	3.88	[3.18; 4.67]	1.0%
Ifeorah, 2017	15	272	5.51	[3.12; 8.93]	0.9%
Ilboudo, 2010	7	115	6.09	[2.48; 12.14]	0.7%
Kfutwah, 2012	51	650	7.85	[5.90; 10.19]	0.9%
Khamis, 2016	1	360	0.28	[0.01; 1.54]	0.9%
Kirbak, 2017	31	280	11.07	[7.65; 15.35]	0.9%
Koneru, 2015	9	252	3.57	[1.65; 6.67]	0.9%
Lennox, 2015	6	150	4.00	[1.48; 8.50]	0.8%
Luuse, 2016	5	208	2.40	[0.79; 5.52]	0.8%
MacLean, 2012	293	3659	8.01	[7.15; 8.93]	1.0%
Makuwa, 2007	109	1186	9.19	[7.61; 10.98]	1.0%
Mamadou, 2012	80	495	16.16	[13.03; 19.71]	0.9%
Mansour, 2012	109	1020	10.69	[8.86; 12.75]	1.0%
Mavenyengwa, 2010	14	418	3.35	[1.84; 5.56]	0.9%
Mbaawuaga, 2008	33	300	11.00	[7.69; 15.10]	0.9%
Metaferia, 2016	21	269	7.81	[4.90; 11.69]	0.9%
Mirambo, 2016	8	211	3.79	[1.65; 7.33]	0.8%
Molla, 2015	17	384	4.43	[2.60; 6.99]	0.9%
Mutagoma, 2017 (2)	486	13121	3.70	[3.39; 4.04]	1.0%
Ndako, 2009	32	200	16.00	[11.21; 21.83]	0.8%
Ndako, 2012	31	180	17.22	[12.01; 23.55]	0.8%
Obi RK, 2006	69	1499	4.60	[3.60; 5.79]	1.0%
Obi SN, 2006	290	10032	2.89	[2.57; 3.24]	1.0%
Ogunlaja, 2015	30	237	12.66	[8.71; 17.57]	0.9%
Okeke, 2012	23	401	5.74	[3.67; 8.48]	0.9%
Okoth, 2006	205	2214	9.26	[8.08; 10.54]	1.0%
Olokoba, 2011	19	231	8.23	[5.02; 12.55]	0.9%
Oluboyo, 2014	6	100	6.00	[2.23; 12.60]	0.7%
Onah, 2007	1	162	0.62	[0.02; 3.39]	0.8%
Onakewhor, 2001	7	320	2.19	[0.88; 4.45]	0.9%
Onwere, 2012	22	810	2.72	[1.71; 4.08]	0.9%
Onwuakor, 2014	25	350	7.14	[4.68; 10.36]	0.9%
Oti, 2018	28	200	14.00	[9.51; 19.59]	0.8%
Ouermi, 2009	26	276	9.42	[6.25; 13.50]	0.9%
Oyinloye, 2016	8	91	8.79	[3.87; 16.59]	0.7%
Pennap, 2011	12	180	6.67	[3.49; 11.36]	0.8%
Pirillo, 2007	10	246	4.07	[1.97; 7.35]	0.9%
Rabiu, 2010	64	1052	6.08	[4.72; 7.70]	1.0%
Rashid, 2014	12	310	3.87	[2.02; 6.66]	0.9%
Sangaré, 2011	103	1139	9.04	[7.44; 10.86]	1.0%
Sidibé, 2001	129	829	15.56	[13.16; 18.21]	0.9%
Strand, 2003	6	60	10.00	[3.76; 20.51]	0.6%
Umare, 2016	22	318	6.92	[4.39; 10.29]	0.9%
Usanga, 2011	8	562	1.42	[0.62; 2.79]	0.9%
Utoo, 2013	55	836	6.58	[4.99; 8.48]	0.9%
Yakasai, 2012	24	303	7.92	[5.14; 11.56]	0.9%
Yohanes, 2016	10	232	4.31	[2.09; 7.78]	0.9%
Zahran, 2010	25	500	5.00	[3.26; 7.29]	0.9%
Zenebe, 2014	12	318	3.77	[1.96; 6.50]	0.9%
**Subgroup prevalence**		**78382**	**6.37**	**[5.58; 7.20]**	**67.1%**
Heterogeneity: I^2 = 94.7% [93.9%; 95.4%], τ^2 = 0.0045, p < 0.0001					
**Overall prevalence**		**104983**	**6.82**	**[6.05; 7.64]**	**100.0%**
**Prediction interval**				**[1.00; 16.96]**	
Heterogeneity: I^2 = 95.8% [95.3%; 96.2%], τ^2 = 0.0064, p = 0					
Test for subgroup differences: χ^2_2 = 23.42, df = 2 (p < 0.0001)					

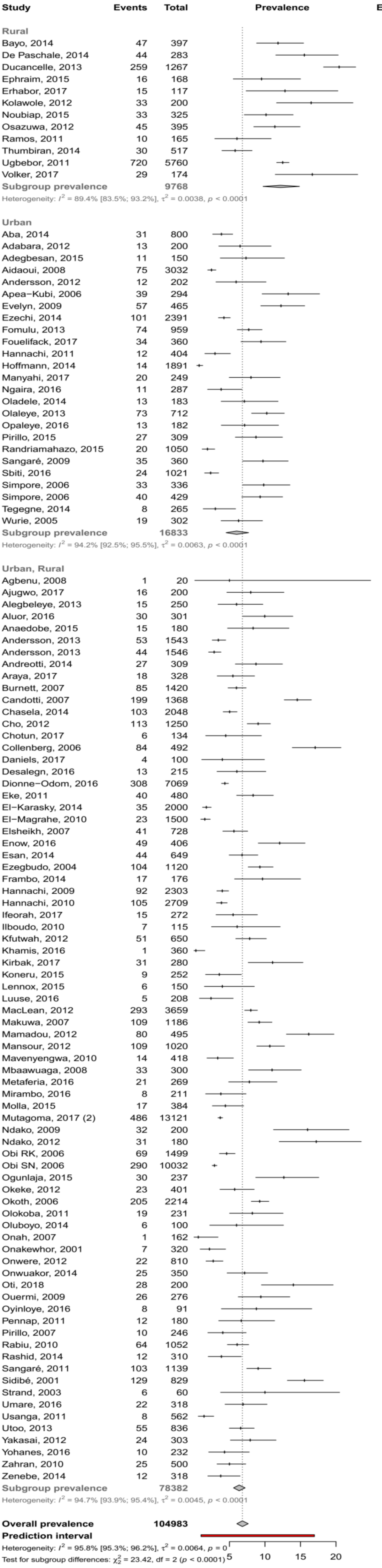

Supplemental Figure 6 : Meta-analysis results for HCV infection prevalence among pregnant women in Africa, by area

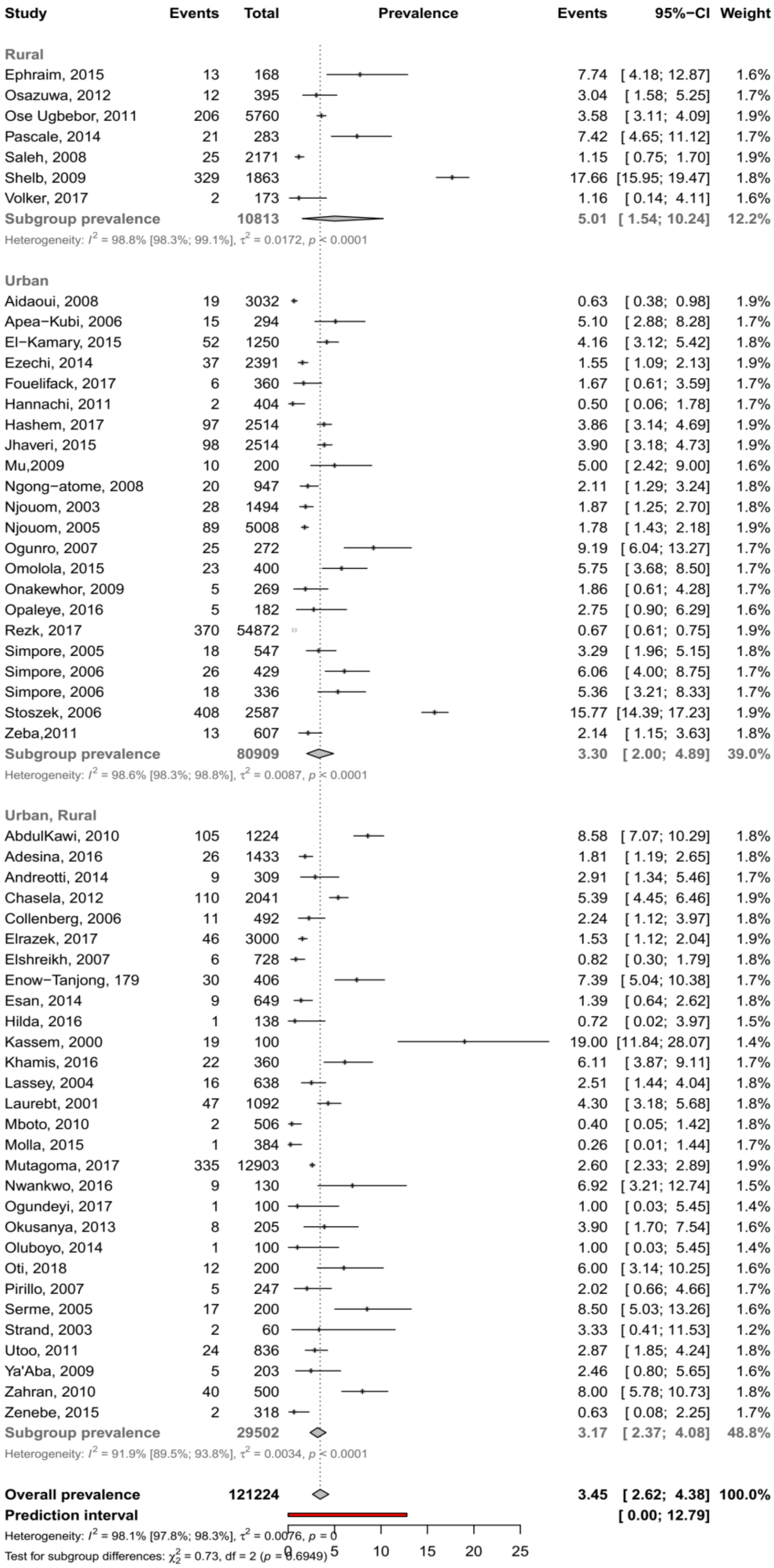

Supplement: Supplementary file 2 — Supplementary Tables and Figures. (PDF 4252 kb) [file 40249_2019_526_MOESM2_ESM.pdf]
